# Optoglycomics: Synthesis and Function of Photochromic Glycoconjugates

**DOI:** 10.1021/jacsau.6c01067

**Published:** 2026-08-28

**Authors:** Guillaume Despras, Thisbe K. Lindhorst

**Affiliations:** † 27091 Université de Toulouse, CNRS UMR 5623, Laboratoire Softmat, 118 Route de Narbonne, 31062 Toulouse Cedex 9, France; ‡ Christiana Albertina University of Kiel, Otto Diels Institute for Organic Chemistry, Otto-Hahn-Platz 3-4, D-24118 Kiel, Germany

**Keywords:** optoglycomics, glycobiology, photopharmacology, photoswitches, azobenzene, lectins, carbohydrate recognition

## Abstract

Optoglycomics describes an emerging research field in which photoswitchable glycoconjugates are employed to probe and control carbohydrate–protein interactions with light. In most cases, azobenzene serves as the photoswitchable chromophore, which can be reversibly isomerized between its *E* and *Z* state, thereby substantially altering the spatial presentation of conjugated glycans. This light-induced conformational change can modulate the affinity of glycan ligands for their receptors and the inhibitory properties of glycosidase and glycosyltransferase inhibitors. Furthermore, glycosylated surfaces, including the cellular glycocalyx, as well as other glycosylated supramolecular assemblies, can be manipulated with the high spatial and temporal precision afforded by light. Although optoglycomics is still in its infancy, the remarkable success of photopharmacology suggests that it has the potential to become a powerful tool for investigating the still not conclusively understood complexity of glycobiology and to inspire new diagnostic and therapeutic strategies for glycan-associated diseases.

## Graphical abstract

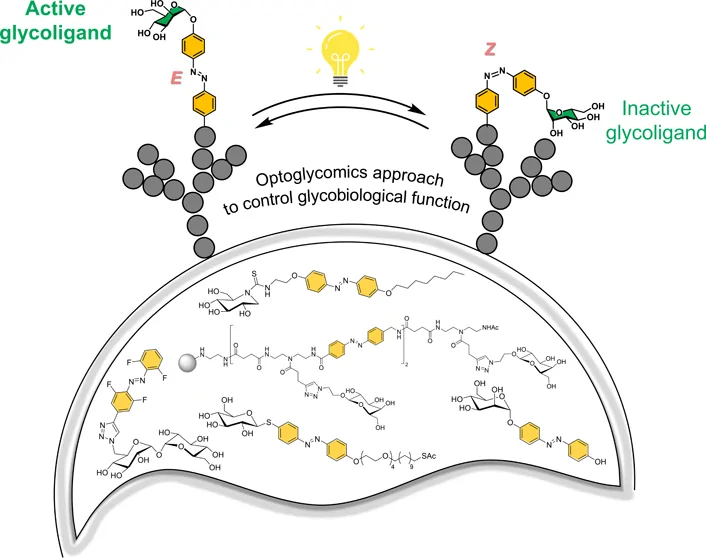

## 1 Introduction

Within biological chemistry, the glycosciences represent one of the major frontiers of the molecular life sciences. Central to this field is the diverse class of carbohydrates and glycoconjugates, which mediate intracellular signaling and, in particular, intercellular communication through the glycocalyx.

The glycocalyx is a dense layer of glycoconjugates that covers the surface of every cell. In eukaryotes, it is a dynamic cellular organelle that regulates recognition, adhesion, signaling, and host–pathogen interactions by engaging a wide range of molecular partners, including receptors, antibodies, enzymes, toxins, microbes, and carbohydrate-binding proteins known as lectins.^1^

The composition and organization of the glycocalyx are highly cell type-specific and change with developmental and physiological state. Alterations of the glycocalyx are a hallmark of numerous diseases, making it an important source of biomarkers and an attractive target for therapeutic intervention.^2^

The complete repertoire of cellular glycans is referred to as the glycome, and its comprehensive analysis constitutes the field of glycomics. Despite remarkable progress, our understanding of glycomics still lags behind genomics and proteomics because glycans exhibit exceptional structural complexity, are not directly genetically encoded, and remain difficult to investigate in their native cellular environment. Moreover, the nanoscale organization of the glycocalyx has long remained inaccessible to experimental analysis.

Recent advances have begun to overcome these challenges. Synthetic glycomimetics, metabolic oligosaccharide engineering, bioorthogonal chemistry, and super-resolution microscopy now enable researchers to manipulate, visualize, and interrogate glycans with unprecedented spatial and temporal resolution, providing new insights into glycocalyx architecture and function.^3^

Among these emerging technologies, photoswitchable glycoconjugates offer a unique opportunity to reversibly control glycan structure and glycan presentation with light. This concept, termed optoglycomics, provides a powerful strategy for correlating glycan organization with biological function. In this Perspective, we introduce the principles of optoglycomics and highlight representative examples demonstrating how photosensitive glycoconjugates are expanding the experimental toolbox of glycobiology.

## 2 The Concept of Optoglycomics

The term optoglycomics was first introduced by the Lindhorst group to describe a research field that employs photoresponsive carbohydrate conjugates to unravel the molecular basis of glycan function and recognition.^4,5^ In optoglycomics, light is used to manipulate the structure and properties of carbohydrate photoswitches, thereby enabling the investigation of key parameters governing glycan organization and carbohydrate recognition. Photoswitches are molecules that can be reversibly switched between two isomeric states, for example an *E* (*trans*) and a *Z* (*cis)* isomer, upon irradiation with light of a distinct wavelength.^6^

The principal advantage of using light to control biological systems lies in its exceptional spatial and temporal precision. Light can be applied noninvasively, while key parameters such as wavelength, intensity, duration and timing of irradiation can be fine-tuned and adjusted. As a result, light can be utilized as a powerful precision tool in biological chemistry and has recently begun to find applications in glycobiology.

Nature utilizes light in many ways, including the regulation of the orientation and motility of diverse organisms. To this end, a variety of photoreceptor proteins (PRPs) have evolved that enable organisms to sense light and respond to the information it conveys (photoresponse).

PRPs typically comprise an apoprotein containing conserved domains that bind light-sensitive cofactors, known as chromophores or photopigments. Depending on the photochemistry of the chromophore, light activation involves either photoreduction or photoisomerization. In both cases, photon absorption induces a conformational change in the apoprotein, thereby initiating signaling pathways that ultimately elicit a cellular photoresponse.^7^

A well-known example is vertebrate vision, in which the photoreceptor protein opsin binds the chromophore 11-*cis*-retinal to form rhodopsin. Photon absorption induces ultrafast photoisomerization of 11-*cis*-retinal to all-*trans*-retinal, triggering conformational changes in rhodopsin that initiate phototransduction and ultimately vision.^8^

The remarkable ability of biological systems to convert light into a biological response through the isomerization of photosensitive molecules inspired the development of optogenetics, which combines genetic engineering with optical control. By relying on genetically encoded photoreceptors, optogenetics has revolutionized neuroscience, enabling the precise manipulation of cells, tissues, and whole organisms while providing unprecedented insights into neural circuits and brain function.^9,10^

The optogenetic framework was subsequently extended by integrating synthetic photoswitches, giving rise to hybrid approaches that enable the interrogation and control of complex biological systems. Trauner and co-workers initially termed this strategy “optochemical genetics,”^11^ and later proposed “photopharmacology” as a more general designation for light-controlled modulation of biological activity.^12^

Suitable photoswitches for incorporation into photoresponsive pharmacophores are those that switch efficiently, exhibit high extinction coefficients and quantum yields, and operate at minimally phototoxic wavelengths. Azobenzene is a prominent example. When conjugated to bioactive molecules, it enables light-controlled modulation of pharmacokinetic and pharmacodynamic properties, a strategy that has been particularly impactful in neuroscience, including applications in visual system modulation.

Compared with conventional chemotherapy, photopharmaceuticals offer the decisive advantage of selective activation or inactivation by light. And in contrast to optogenetic approaches, photopharmacology does not rely on genetic engineering or gene therapy, thereby significantly increasing its potential for clinical applications.

This brief historical overview, spanning from photoreceptor proteins to optogenetics and photopharmacology, highlights the conceptual foundation of photochemical approaches in biological systems and underscores the potential of optoglycomics. Analogously, synthetic photoswitchable glycoconjugates in solution, on surfaces, or within the glycocalyx may emerge as powerful tools in the glycosciences, exploiting the unique spatiotemporal control afforded by light. Given the growing recognition of the glycocalyx as a therapeutic target, optoglycomics may enable new functional, diagnostic, and therapeutic strategies.

Figure 1 summarizes the principal dimensions of optoglycomics, exploiting photoswitchable glycomimetics that can be reversibly interconverted by light. The examples shown employ azobenzene as the representative chromophore because it is the most widely used photoswitch in the field (see subsequent sections). Although not all concepts have yet been fully realized, they illustrate the breadth of opportunities offered by optoglycomics.

**Figure 1:**
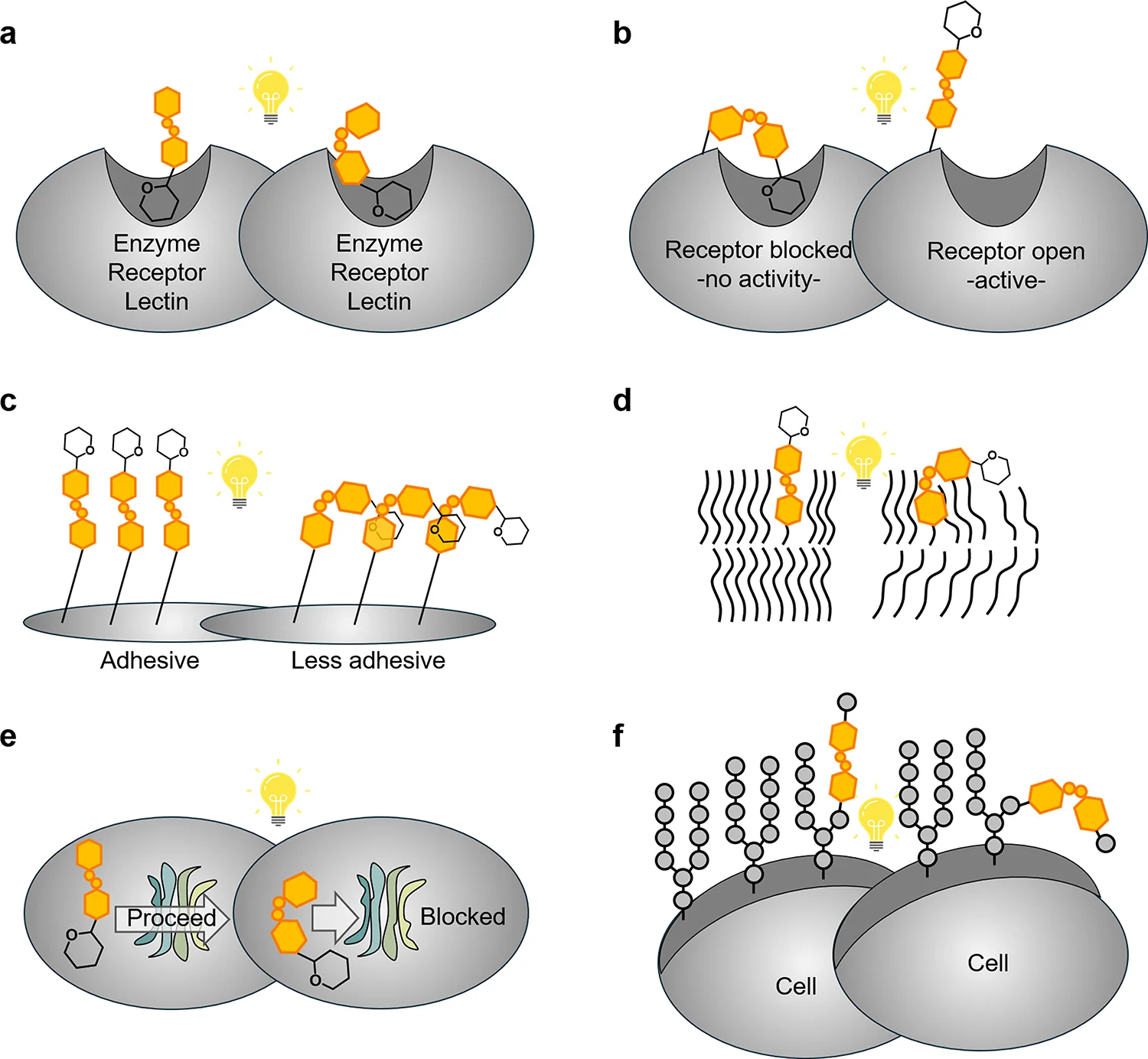
Dimensions of Optoglycomics. (a) Photocontrol of ligand/substrate affinity. (b) Photocontrol of receptor binding activity. (c) Modulation of the adhesiveness of surfaces. (d) Phase transition in lipid mono- and bilayers/supramolecular systems. (e) Photocontrol of metabolism. (f) Glycocalyx remodeling through metabolic engineering.

Photoswitchable glycans have been employed as lectin ligands and substrates for glycan-processing enzymes (Figure 1a). Because the two photoisomers differ in geometry and physicochemical properties, they often exhibit distinct binding affinities or enzymatic reactivities. Consequently, one isomer may inhibit receptor binding or enzyme activity, whereas irradiation restores function by generating the alternative isomer.

Glycophotoswitches can also be covalently installed on proteins using affinity-guided labeling strategies (Figure 1b).^13^ Light-induced isomerization then reversibly occludes or exposes binding sites, enabling optical control of protein function. At the supramolecular level, incorporation into self-assembled monolayers permits reversible switching between adhesive and weakly adhesive states, thereby modulating carbohydrate-dependent bacterial adhesion (Figure 1c). A related strategy can be implemented in living cells through metabolic oligosaccharide engineering to reversibly remodel glycocalyx architecture (Figure 1f). Ultimately, such approaches may become applicable in vivo and help elucidate how glycan organization governs molecular recognition and downstream biological function.

Another emerging direction is the optical control of glycan metabolism (Figure 1e). Photoswitchable metabolic intermediates or enzyme substrates could enable precise temporal control of glycan biosynthesis, for example during antibody glycosylation. Optoglycomics also extends to biological membranes (Figure 1d). Incorporation of photoswitchable glycolipid mimetics provides a noninvasive means to reversibly induce and study membrane phase transitions and other supramolecular reorganizations that are central to cellular function.

Collectively, photoswitchable glycomimetics enable optical control of glycan function across multiple levels of biological organization, from molecular recognition and supramolecular assemblies to living cells and, potentially, whole organisms, offering new opportunities for both fundamental glycobiology and future diagnostic and therapeutic applications.

## 3 Focus and Scope of this Account

In this Perspective, we focus on recent advances in optoglycomics involving three classes of carbohydrate-binding proteins, namely lectins, glycosidases, and the transmembrane glycoprotein CD1d (cluster of differentiation 1d). In addition, we have included a section on photoswitchable glycolipid mimetics employed to study the dynamics of glycosylated surfaces and membrane models.

We highlight representative examples in which photoswitchable glycoconjugates have been used to interrogate biological function. Collectively, photosensitive glycoconjugates are discussed across different levels of structural complexity, ranging from small soluble photoswitches to multivalent constructs and extending to supramolecular assemblies, lipid mono- and bilayers, self-assembled monolayers, and ultimately cellular surfaces.

The following subchapters provide concise introductions to the carbohydrate-specific proteins and membrane models that are the focus of this account.

### 3.1 Lectins

Lectins are carbohydrate-binding proteins found in virtually all forms of life, including mammals, plants and pathogens. They play key roles in cell recognition, signaling, intracellular trafficking, and modulation of the immune response. In particular, lectins mediate the adhesion of pathogens and cancer cells to host tissues and are therefore extensively studied both to decipher the molecular determinants of carbohydrate–protein recognition and to develop therapeutic strategies against infection.

Among the most investigated lectins are plant lectins such as concanavalin A (ConA) and peanut agglutinin (PNA), as well as bacterial adhesins such as FimH (the type 1 fimbrial lectin) and the *Pseudomonas aeruginosa* lectins LecA and LecB. While plant lectins are valuable model systems for studying carbohydrate–protein interactions and also hold significant therapeutic potential, particularly in oncology, bacterial lectins serve adhesion to host cells which is mediated by adhesive appendages on the surface of bacterial cells called fimbriae.^14^ FimH is expressed at the tip of *Escherichia coli* type 1 fimbriae and specifically recognizes α-d-mannosyl residues. Pathogenic *E. coli* strains exploit strong, multivalent FimH–mannose interactions with mannosylated epithelia to colonize the urinary tract and cause severe infections. Another opportunistic pathogen, *P. aeruginosa*, uses LecA and LecB to adhere to tissues and colonize the host by forming highly resilient biofilms, potentially leading to systemic infection and sepsis. While LecA displays a high specificity for d-galactosyl residues, LecB preferentially binds l-fucosyl and d-mannosyl epitopes. Because of their central role in colonization and infection, bacterial lectins are considered critical virulence factors and are prime targets for the development of antiadhesive antibacterial therapies as alternatives to conventional antibiotics.^15,16^ The structures and carbohydrate specificities of these lectins are now well characterized, making them powerful model receptors for dissecting specific interactions with the glycocalyx.

In this context, optoglycomics offers new opportunities to investigate lectin–glycan interactions. Photoswitchable lectin ligands, in which light modulates glycan presentation, provide an attractive strategy to probe and control lectin–glycan recognition within the glycocalyx and to develop next-generation antiadhesive agents.

### 3.2 Glycosidases

Glycosidases are enzymes that cleave glycosidic bonds in oligosaccharides and glycoconjugates, and are essential for digestion, lysosomal degradation of glycoconjugates, and glycoprotein processing.^17^ Defects or dysregulation of specific glycosidases cause lysosomal storage disorders (LSDs), contribute to cancer progression, viral infection, and other metabolic diseases.^18,19^

Numerous small-molecule glycosidase inhibitors, often sugar mimics such as iminosugars, carbasugars, and carbaglycosylamines, have been developed both as mechanistic probes and therapeutic agents. Key applications include the treatment of LSDs, cancer, diabetes, and viral infections, either by reducing substrate accumulation or by modulating glycoprotein processing. Pharmacological chaperones (PCs) are typically competitive glycosidase inhibitors that bind misfolded mutant enzymes in the endoplasmic reticulum, stabilize folding, and promote lysosomal trafficking; they must subsequently dissociate to restore enzymatic function.^18^ These compounds are particularly important for neuropathic LSDs (e.g., Gaucher, Fabry, and GM1-gangliosidosis), where biologics cannot cross the blood–brain barrier. Therapeutic strategies include optimized iminosugars and carbasugars, as well as pH-responsive or reversibly covalent ligands that self-inactivate in the lysosome.^20–22^

Collectively, glycosidase inhibitors already play a major role as pharmacological chaperones and in diagnostic and therapeutic applications. In this context, optoglycomics offers a means to modulate glycosidase activity with light. Photoswitchable glycosidase substrates enable reversible, light-controlled tuning of inhibition and chaperone activity, with promising implications for precision intervention in lysosomal and other glycosidase-related disorders.

### 3.3 CD1d-NKT

CD1d is a transmembrane glycoprotein and an MHC class I–like antigen-presenting molecule that binds lipid and glycolipid antigens and presents them to invariant natural killer T (iNKT) cells and other CD1d-restricted T cells.^23,24^ Its hydrophobic binding groove accommodates diacylglycerols, phospholipids, glycosphingolipids, and related lipids, which are recognized by semi-invariant or diverse T cell receptors (TCRs) on NKT cells.

Through the formation of ternary complexes involving CD1d, lipid antigens, and TCRs, iNKT cells rapidly produce large amounts of cytokines (e.g., IFN-γ, IL-4, IL-13, and TNF-α), thereby linking innate and adaptive immunity and shaping immune responses against infections, tumors, and autoimmune disorders.^25,26^ CD1d expression on antigen-presenting cells, B cells, T cells, and other tissues further underlines its role in cancer progression, infectious disease control, arthritis, and humoral immunity.^27,28^

Therapeutically, synthetic glycolipids such as α-galactosylceramide (α-GalCer/KRN7000) and related analogues are used to activate or modulate iNKT cells via CD1d in cancer immunotherapy, infectious disease models, and vaccine development. However, clinical translation is limited by issues such as mixed Th1/Th2 polarization, anergy induction, and rapid clearance.^29–31^

In this context, optoglycomics offers a strategy to control antigen presentation with light. Photoswitchable CD1d ligands, including modified analogues of α-GalCer, enable spatiotemporal control of iNKT cell activation and cytokine release, thereby providing a powerful approach to interrogate and modulate CD1d-mediated immune responses.

### 3.4 Glycosylated Membrane Models

Glycolipids are important constituents of the glycocalyx and fulfill diverse structural and functional roles in biological membranes. Because their impact on membrane dynamics and function is difficult to investigate directly in living cells, simplified membrane models, including Langmuir monolayers, Langmuir–Blodgett films, and supported lipid bilayers, have become valuable experimental platforms.^32^ These systems allow the study of key aspects of membrane organization and restructuring under well-defined conditions.

Incorporation of photoswitchable surfactants into membrane models enables to study the influence of surfactant geometry on membrane properties such as lateral packing, membrane fluidity, bending rigidity, lateral pressure, layer thickness, lipid topology, and mesophase behavior. Depending on the molecular design, photoswitching can induce reversible membrane restructuring and transitions between different liquid-crystalline phases, providing fundamental insights into the relationship between glycolipid structure and membrane function.^33^

In this context, optoglycomic approaches provide a means to investigate such changes through azobenzene-based glycolipid mimetics incorporated into Langmuir monolayers and related membrane models. In combination with Langmuir isotherms and X-ray scattering techniques, these studies have established a powerful methodological framework for correlating molecular photoisomerization with structural alterations in glycosylated membrane models.

## 4 The Unique Properties of Azobenzene Photoswitches

Optoglycomics relies on photochromic molecules that can be reversibly interconverted between two states by irradiation with light of different wavelengths. Photoisomerization induces pronounced changes in molecular geometry, polarity, and other physicochemical properties,^34^ thereby enabling precise spatiotemporal control over molecular recognition, self-assembly, and biological function.^35^

Among the many classes of molecular photoswitches,^36–39^ azobenzene (AB) has emerged as the most widely used because of its favorable photochemical properties and synthetic versatility. Upon irradiation, azobenzene undergoes reversible isomerization between the thermodynamically stable, elongated *E* isomer and the bent *Z* isomer, reducing the *para*-carbon distance from approximately 9 Å to 5.5 Å (Figure 2).^40^ This well-defined conformational change is frequently exploited to modulate receptor–ligand interactions, molecular conformation, and the spatial presentation of bioactive ligands.

**Figure 2:**
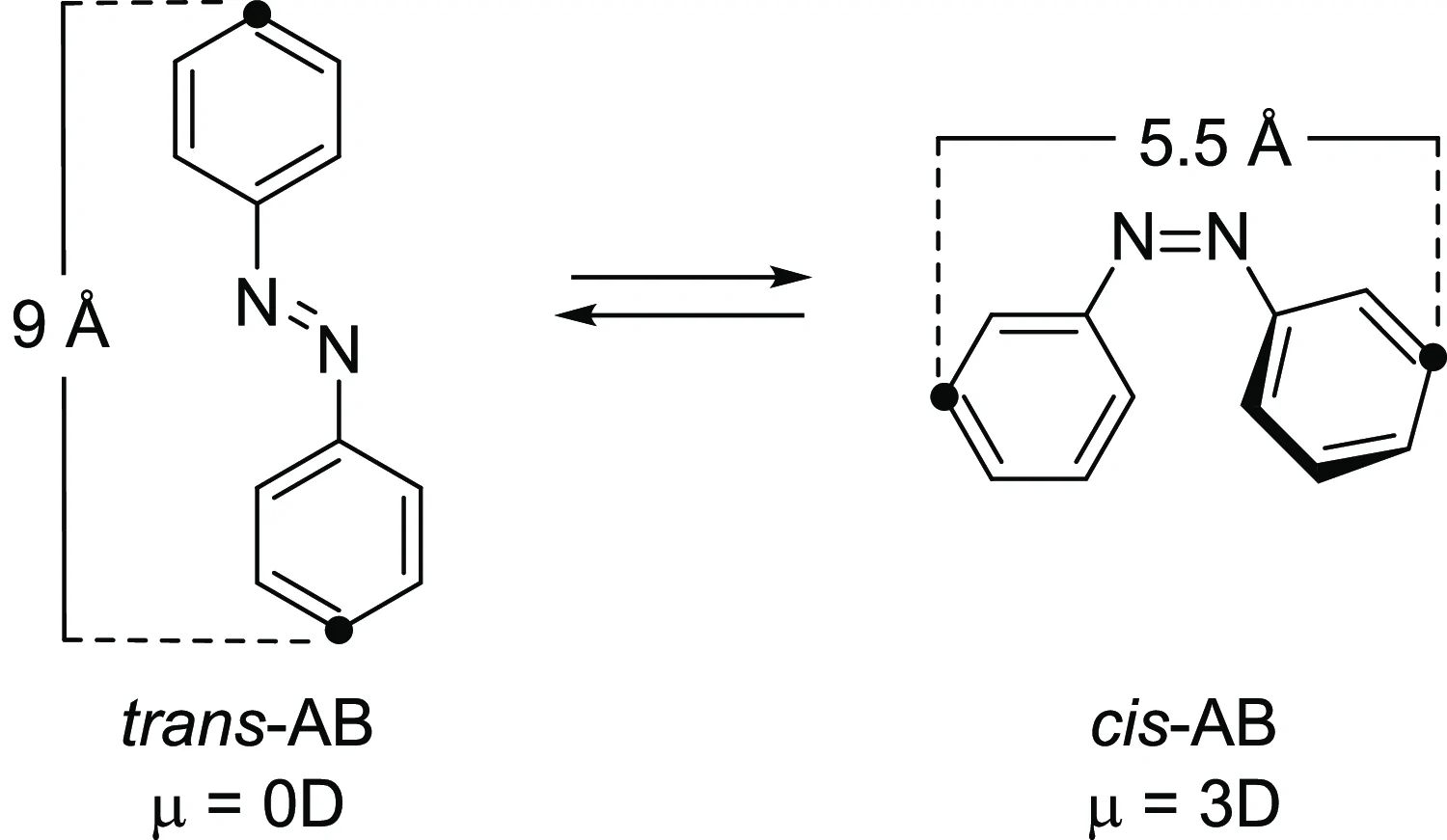
Azobenzene in its *E* and *Z* state, differing in the dipole moment μ and the distance of the *para*- and *para’*-carbon atoms (highlighted as dots).

Azobenzenes further combine high photoisomerization efficiency, excellent fatigue resistance, good aqueous stability, and tunable photophysical properties.^6,41^ Chemical substitution enables systematic adjustment of water solubility, photostationary state (PSS), and thermal *Z* → *E* relaxation kinetics (thermal half-life τ_1/2_), allowing photoswitches to be tailored for specific applications.^42^ Photoisomerization also alters the dipole moment and hydration shell of the molecule, which can influence interactions with biological targets.^43^

The *E* → *Z* isomerization of the N=N (diazene) double bond in azobenzene derivatives occurs upon exposure to UV light (300–400 nm) but also under mechanical stress or electrical stimulation.^41,44^ Reisomerization to the *E*-isomer can be affected by absorption of blue light (∼450 nm) but this isomerization is not complete due to the partial overlap in the UV/vis spectra of the *E* and *Z* isomers of azobenzenes. The thermal *Z* → *E* relaxation, on the other hand, is quantitative.

A major limitation of classical azobenzenes is that their photoisomerization relies on UV or blue light, which exhibits limited tissue penetration and may cause phototoxicity. Accordingly, extensive efforts have focused on bathochromically shifting azobenzene absorption toward the red or far-red region by modifying either the azobenzene scaffold or the linker connecting it to glycans.^45^ These strategies improve tissue penetration and expand the applicability of azobenzene glycoconjugates in in vivo settings, opening new opportunities for biological applications.

Taken together, azobenzenes (ABs) are versatile molecular scaffolds for the design of photosensitive glycosides and glycoconjugates that enable optical control over glycan presentation, spatial organization, and multivalent ligand display. Their broad synthetic accessibility has further facilitated the development of diverse azobenzene glycoconjugates (ABGs), as discussed in the following chapter.

## 5 Design of Azobenzene Glycoconjugates—A Synthetic Toolbox

### 5.1 The Beginnings

In the early 2000s, Jayaraman and co-workers made seminal contributions to what is now considered the emerging field of optoglycomics by exploring azobenzene-based photoswitchable cluster glycosides for multivalent interactions with lectins (Figure 3).^46,47^ In these studies, multivalent scaffolds featuring an azobenzene core functionalized with mannosyl or lactosyl glycoligands were used to selectively target the plant lectins concanavalin A (ConA) and peanut agglutinin (PNA), respectively. Isothermal titration calorimetry (ITC) revealed cooperative binding that was significantly enhanced in *Z*-rich photostationary states of the azobenzene cluster glycosides.

**Figure 3:**
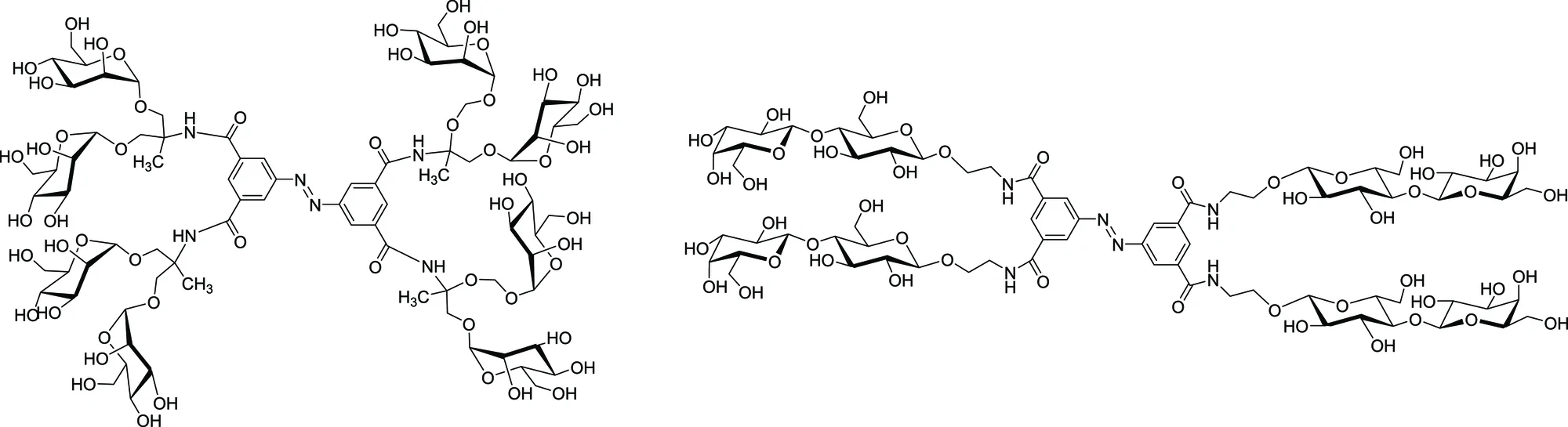
Representative examples of the first ABGs to test lectin affinity in dependence of the isomeric state of the azobenzene core.

The early studies by Jayaraman and co-workers demonstrated that the spatial arrangement of glycoligands is a key determinant in carbohydrate–protein interactions, establishing the foundation for the use of azobenzene glycoconjugates (ABGs) as light-responsive tools to control carbohydrate orientation and presentation.

Since these pioneering reports, ABGs have emerged as versatile molecular probes for interrogating carbohydrate–protein interactions, driven by the pronounced structural differences between the *E* and *Z* isomers of the azobenzene scaffold. Many of the targeted proteins are of therapeutic relevance and considered druggable, motivating extensive efforts to exploit ABGs as photocontrollable agonists and antagonists within the broader context of photopharmacology.

In optoglycomics, structure and function are closely intertwined, making molecular design and synthetic strategy central to the development of azobenzene glycoconjugates (ABGs). Their architecture and mode of glycan presentation are tailored to the target protein and intended application, giving rise to a diverse repertoire of synthetic strategies for coupling carbohydrates to azobenzene scaffolds.

As illustrated in Figure 4, the photoswitch can be attached directly to the carbohydrate or introduced through a linker. Common motifs include azobenzene *O*-glycosides and anomeric thioglycosides (Figure 4a), the latter of which can be oxidized to sulfones to tune the electronic and photophysical properties of the ABG. Alternatively, azobenzene may be coupled through functionalized linkers at the anomeric position or other sites of the carbohydrate scaffold using complementary functional groups (FG^1^/FG^2^; Figure 4b,c). This modular strategy also facilitates incorporation of multiple azobenzene units with orthogonal switching behavior.

**Figure 4:**
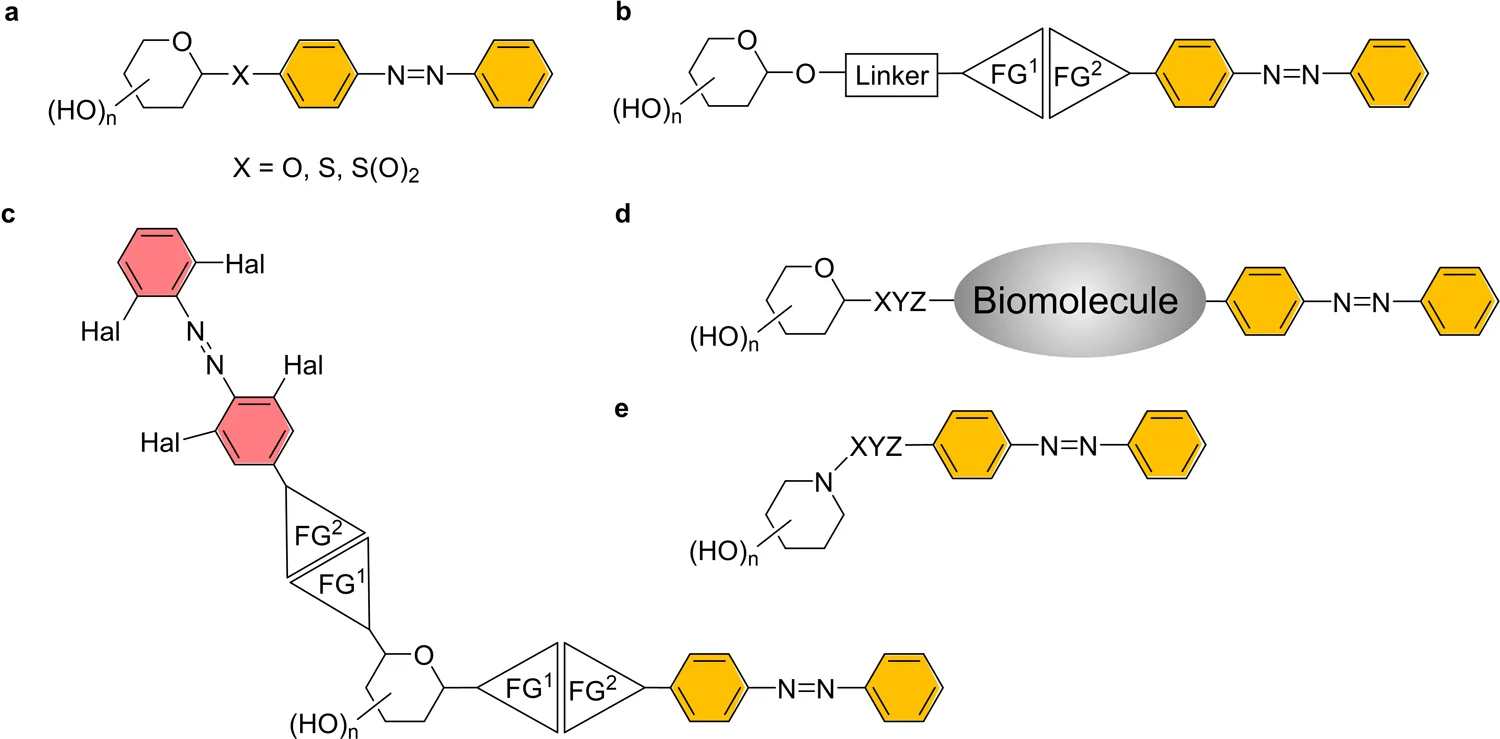
Different architectures of ABGs. See text for details. FG: functional group.

More elaborate designs integrate azobenzene into glycopeptide or glycolipid mimetics, in which the glycan is linked to a noncarbohydrate scaffold through diverse chemistries (Figure 4d). Structural diversity can be further expanded by incorporating the photoswitch into azasugars, for example through attachment to the endocyclic nitrogen via thiourea or related linkages (XYZ, Figure 4e).

The following sections summarize the principal synthetic strategies for constructing ABGs with tailored photophysical and biological properties, alongside key references for each approach. These strategies broadly follow the classification outlined in Figure 4. We first discuss approaches for achieving bathochromic shifts in azobenzene glycoconjugates.

### 5.2 Red-Shifting of Azobenzenes

For biological applications, particularly in vivo, it is highly desirable to shift azobenzene photoisomerization from the UV into the visible or red spectral region, where light penetrates tissue more efficiently and causes less photodamage.^45^ Fortunately, the electronic and photochemical properties of azobenzenes can be systematically tuned through substitution of the aromatic rings.

Early strategies employed electron-donating or push–pull substitution patterns to red-shift absorption. Although effective, these modifications often increase spectral overlap between the *E* and *Z* isomers and accelerate thermal relaxation, thereby compromising photoswitching performance.^41^ In contrast, tetra-*ortho*-substituted azobenzenes, particularly methoxy-, chloro-, and fluoro-substituted derivatives achieve efficient visible-light switching while maintaining favorable photostationary states and high thermal stability. Among these, tetra-*ortho*-fluoro azobenzenes have become especially valuable because they combine excellent switching efficiencies with long-lived *Z* isomers.^48,49^

By comparison, substitution in the *meta* position generally exerts only modest effects on absorption properties and thermal stability relative to *ortho*- and *para*-substitution. Beyond classical azobenzenes, heteroaryl azobenzenes and diazocines further expand the photochemical toolbox by providing visible-light responsiveness and improved switching characteristics but are not discussed in this Perspective.^50^

Importantly, the nature of the linkage between the glycan and the azobenzene scaffold can also influence the photophysical properties of the resulting ABGs. In particular, thioglycosidic linkages induce a pronounced bathochromic shift relative to their *O*-glycosidic counterparts (Figure 5).

**Figure 5:**
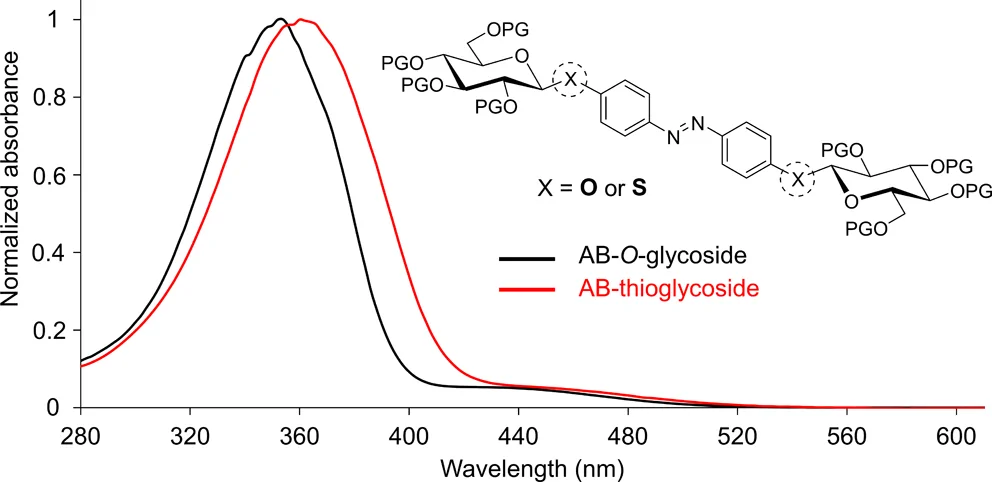
Overlay of representative UV–vis spectra of AB *O*-glycosides (black line) versus AB thioglycosides (red line). The latter exhibit a significant red-shift in absorbance, due to the electron-donating effect of the thioglycosidic linkage. As an example, the unpublished UV/vis spectrum (black) of an *O*-acetylated bis-*O*-glucoside^51^ and the published UV/vis spectrum (red) of an *O*-acetylated bis-*S*-glucoside^52^ are depicted. PG: protecting group.

Collectively, these molecular engineering strategies have transformed azobenzene from a UV-responsive photoswitch into a versatile platform for visible-light-controlled glycoconjugates, substantially expanding the experimental and therapeutic opportunities of optoglycomics.

### 5.3 Direct Conjugation of Glycan and AB

#### 5.3.1 AB *O*-Glycosides

When the carbohydrate ligand is attached directly to the aromatic scaffold through a glycosidic bond, *E*/*Z* photoisomerization produces the largest change in glycan orientation. This architecture is particularly advantageous for soluble ligands targeting lectins and glycosidases, where subtle reorientation of the carbohydrate epitope within the binding site must be efficiently transmitted by the azobenzene hinge.^53–55^

Accordingly, direct glycosylation of *para*-hydroxyazobenzene derivatives has become the preferred strategy for the synthesis of such ABGs. In addition to their straightforward architecture, azobenzene *O*-glycosides exhibit favorable photophysical properties, including high photoisomerization efficiencies and slow thermal relaxation of the metastable *Z* isomer, making them well suited for applications as photoswitchable protein ligands.^56^

Most reported syntheses rely on glycosyl trichloroacetimidates as donors and monohydroxyazobenzene (MHAB) and dihydroxyazobenzene (DHAB) as glycosyl acceptors (Scheme 1, compounds **1** and **2**). Among these, *p,p*′-DHAB is particularly attractive because further functionalization at the *para*′ position provides access to both symmetrical and unsymmetrical bis-glycosides.^53,57,58^ Despite this synthetic flexibility, glycosylation of *p,p*′-DHAB remains challenging, often affording variable yields together with poor regio- and stereoselectivities despite considerable efforts to improve the reaction.^51^

**Scheme 1:**
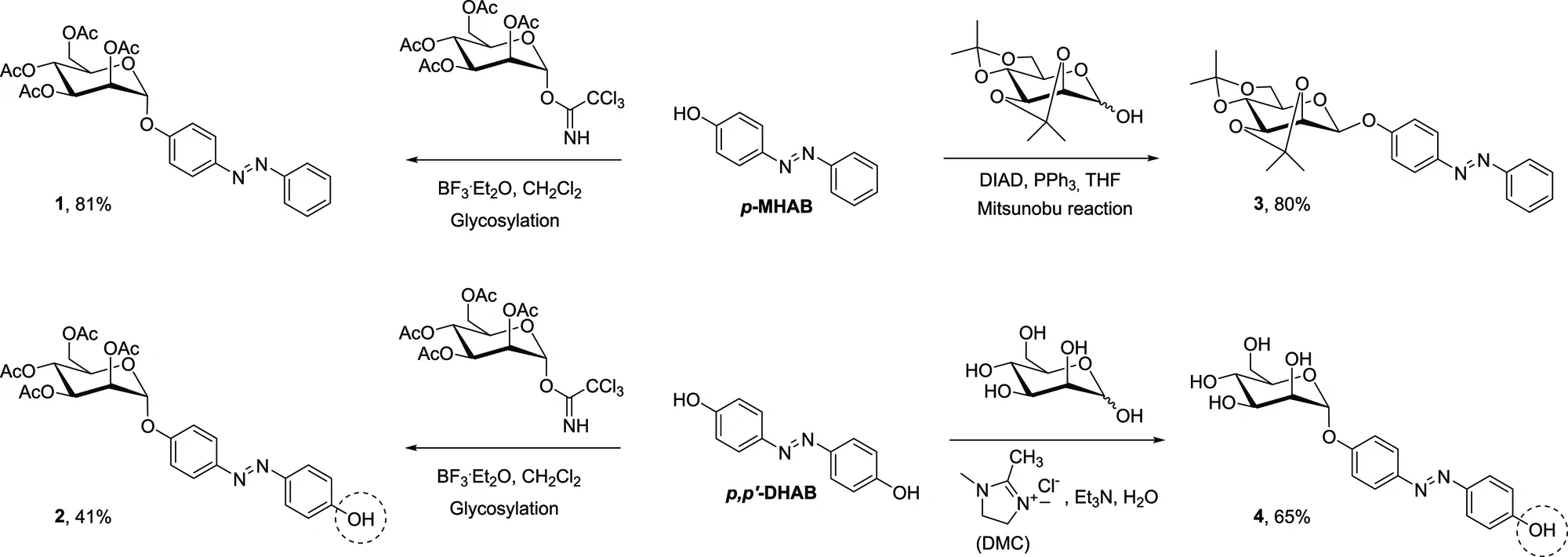
Synthesis of Azobenzene *O*-Glycosides Using Various Methods

To circumvent the use of activated glycosyl donors such as glycosyl imidates, alternative glycosylation strategies have been developed for the synthesis of ABGs. Among these, the Mitsunobu reaction has proven particularly useful, enabling the coupling of MHAB or DHAB with protected or unprotected carbohydrates (Scheme 1).^59^ For example, isopropylidene-protected d-mannose afforded the corresponding β-mannoside **3** in high yield, whereas unprotected d-mannose yielded exclusively the α-anomer, albeit less efficiently. The Mitsunobu reaction can also be applied to the selective functionalization of primary hydroxyl groups in carbohydrates.^60^

More recently, Xie and co-workers reported the direct glycosylation of DHAB with unprotected sugars in water using 2-chloro-1,3-dimethylimidazolinium chloride (DMC, Shoda’s reragent) as a dehydrating reagent.^61^ This method furnished a series of mono- and bis-AB glycosides in low to moderate yields but with excellent stereoselectivity. Moreover, allyl- and propargyl-functionalized ABGs, exemplified by mannoside **4** (Scheme 1), were obtained in only two steps, providing versatile intermediates for further derivatization.

Although these methods provide efficient access to AB glycosides, the synthesis of red-shifted ABGs along these lines remains considerably more challenging. Such compounds typically require *ortho*-substituted azobenzene scaffolds bearing additional hydroxyl groups, which are not readily accessible and generally require multistep syntheses. Nevertheless, late-stage *ortho*-chlorination of azobenzenes can be achieved by palladium-catalyzed C–H activation,^62^ to furnish red-shifted ABGs;^63^ their photoisomerization efficiencies, however, are lower than those of the corresponding parent compounds.

#### 5.3.2 AB Thioglycosides

Seeking a general and efficient method for directly linking azobenzene (AB) motifs to glycosyl residues, our group developed a palladium-catalyzed thioarylation, also known as the Buchwald–Hartwig–Migita cross-coupling.^64^ Compared with *O*-glycosylation, this approach offers several advantages. It employs readily accessible coupling partners, namely halo- (iodo- or bromo-) substituted AB derivatives (Scheme 2, compounds **5–7**), including red-shifted photoswitches, and glycosyl thiols (Scheme 2, compounds **8** and **9**). The reaction proceeds under mild conditions using the third-generation Buchwald precatalyst XantPhos Pd G3 and is compatible with a broad range of functional groups and solvents. Moreover, it is stereoretentive, particularly when anomeric thiols are employed.

**Scheme 2:**
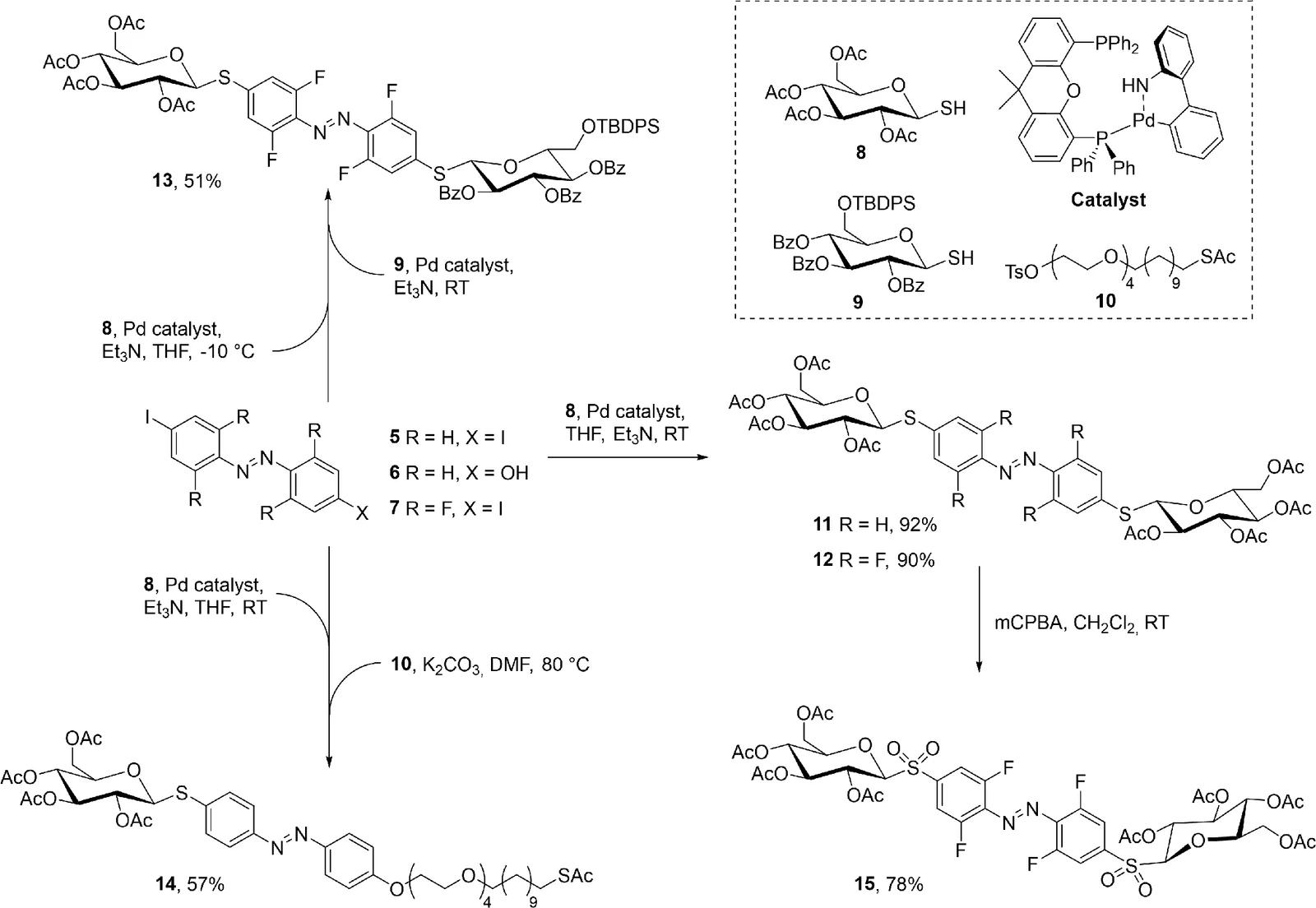
Synthesis of Symmetrical and Unsymmetrical ABGs through Buchwald–Hartwig–Migita Cross-Coupling

In 2022, we demonstrated the broad substrate scope of this transformation.^52^ Symmetrical AB glycoconjugates such as **11** and **12** were obtained in excellent yields, whereas unsymmetrical derivatives (**13** and **14**) were prepared in a one-pot procedure, likewise in good yields (Scheme 2). Beyond its synthetic versatility, the method affords AB thioglycosides with attractive photochromic properties. The sulfur linkage also enables systematic tuning of the absorption and thermal relaxation properties through oxidation to the corresponding sulfoxides or sulfones, most notably by inducing bathochromic shifts. This versatility is exemplified by the orthogonal photoswitching of mixtures containing sulfide **11** and sulfone **15** (Figure 6).

**Figure 6:**
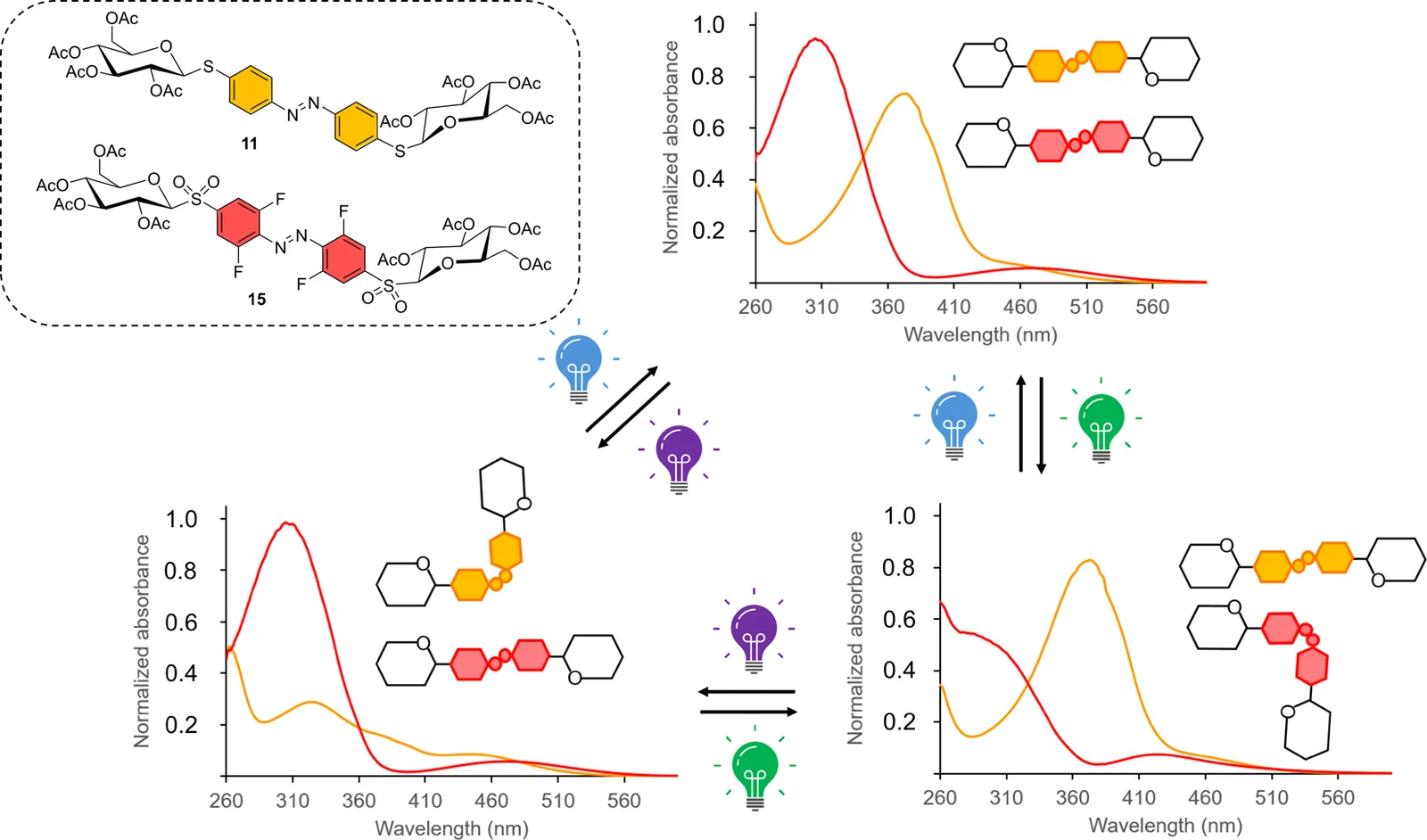
Orthogonal photoswitching of AB thioglycosides derivatives in 1:1 solution mixture. The UV–vis spectra show the thioglycoside **11** (orange line) and the corresponding sulfone **15** (red line) at PSS reached after UV-, green- or blue-light irradiation.

#### 5.3.3 Direct Connection at Functionalized Carbohydrate Ring Positions

Other approaches have been implemented to directly conjugate AB and glycan moiety. For example, 6-azido-6-deoxy-trehalose (**16**) was coupled with propargylated tetrafluoro azobenzene **17** in a copper-catalyzed azide–alkyne cycloaddition (CuAAC) to yield the ABG **18**, which can be taken up by Mycobacteria (Scheme 3).^39^ On the other hand, l-fucose could be converted into its aminomethyl *C*-glycoside (**19**) by a Henry condensation reaction to allow the synthesis of ABGs with amide (**20**) or sulfonamide (**21**) bond linkage (Scheme 3).^65^

**Scheme 3:**
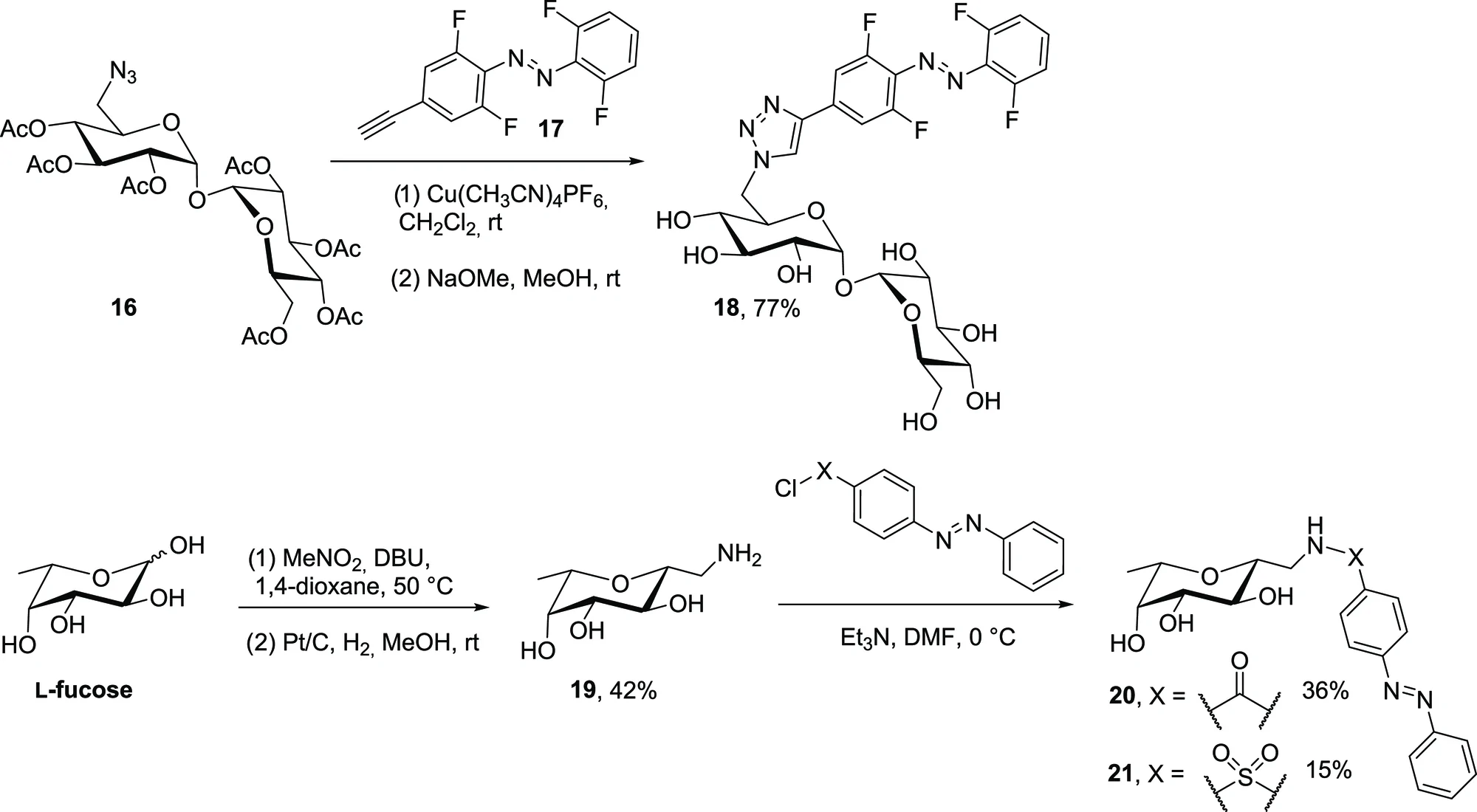
Access to ABGs via Click Chemistry (CuAAC) and Amide or Sulfonamide Conjugation

### 5.4 Late Stage Azo Group Formation

As an alternative to coupling a preformed azobenzene scaffold with a glycan, ABGs can also be synthesized by late-stage formation of the azo bond, for example via the Mills reaction. This strategy is particularly attractive for the preparation of unsymmetrical ABGs and 1,2-*cis*-configured azobenzene glycosides, which are difficult to access by direct glycosylation. Using this approach, Lindhorst and co-workers synthesized α-d-azobenzene glucosides **23** and **24** from readily available *p*-aminophenyl α-d-glucoside **22** and nitrobenzene derivatives (Scheme 4).^56^

**Scheme 4:**
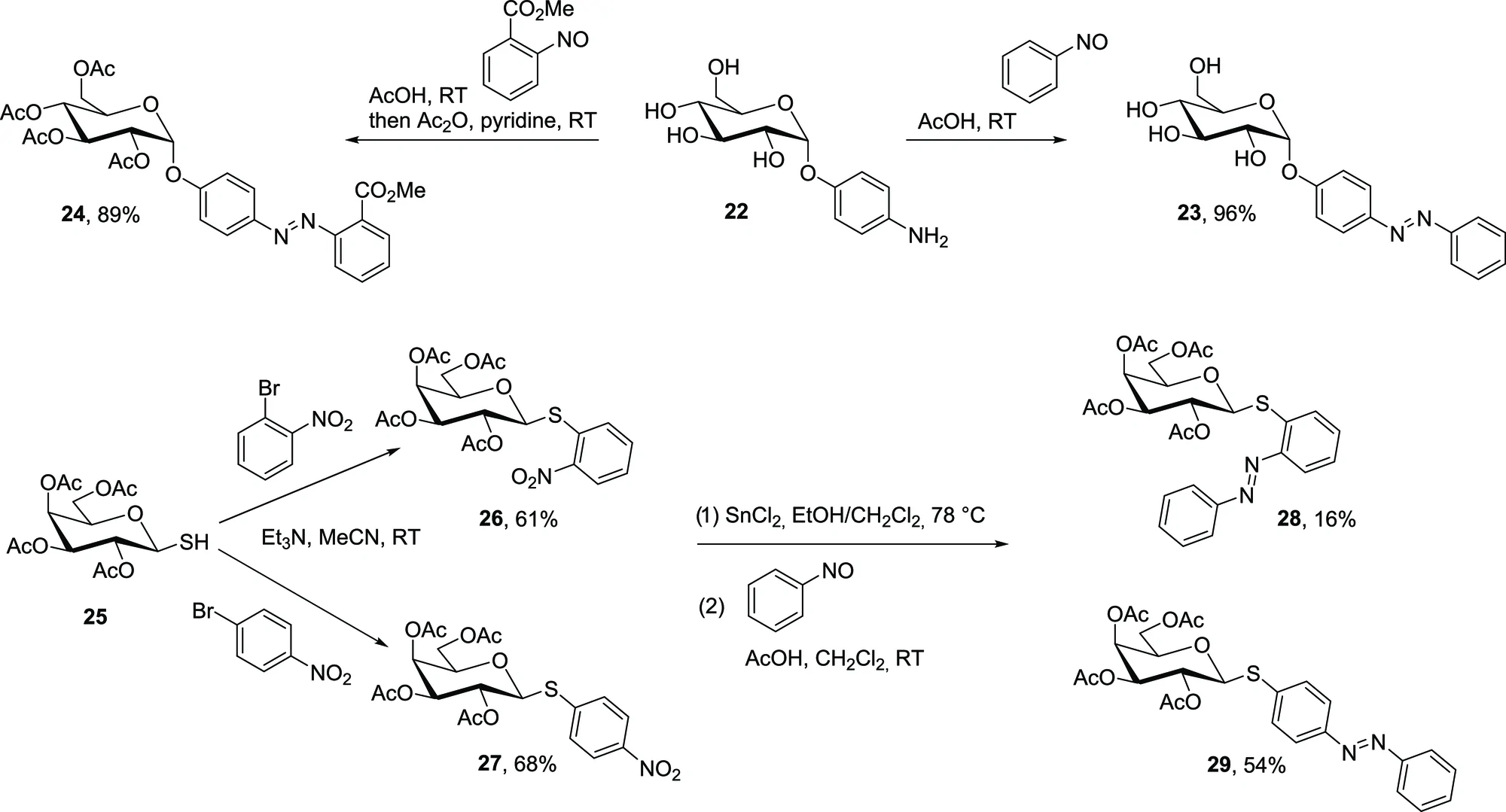
Synthesis of ABGs via Late Stage Formation of the Azo Bond

König and co-workers likewise employed the Mills reaction to prepare azobenzene thiogalactosides. They first coupled the anomeric thiol **25** with bromonitrobenzene via nucleophilic aromatic substitution (S_N_Ar, products **26** and **27**), followed by tin­(II) chloride-mediated reduction to generate the aniline precursor for the subsequent Mills coupling leading to **28** and **29** (Scheme 4).^66^

### 5.5 Linker-Mediated Conjugation of Glycans and AB

In contrast to linker-free architectures, many ABGs are designed such that the azobenzene unit is positioned remotely from the carbohydrate epitope through a molecular linker. This architecture is characteristic of glycooligomers, azobenzene-functionalized glycolipid mimetics, and amphiphilic systems that self-assemble into micelles, vesicles, or hydrogels.^67–70^ In these systems, photoisomerization primarily modulates molecular packing, supramolecular organization, and the local microenvironment rather than directly altering the orientation of the carbohydrate within a protein binding site.

An important example is represented by photoswitchable CD1d-NKT ligands based on galactosylceramides, in which photoisomerization selectively changes the geometry of the lipid chain bound to CD1d, while the galactose headgroup continues to engage the NKT cell receptor.^71^ Likewise, ABGs targeting glycosidases have been prepared with aliphatic spacers separating the carbohydrate from the azobenzene unit. In these systems, linker length and flexibility influence both enzyme recognition and the thermal stability of the *Z* isomer.^72^

Remote incorporation of azobenzene is frequently achieved by copper-catalyzed azide–alkyne cycloaddition (CuAAC), particularly in the synthesis of photoswitchable glycolipids and glycooligomers.^69,73^ Typically, azide-functionalized carbohydrates (Scheme 5, **30** and **32**) are coupled with alkyne-substituted azobenzene derivatives (Scheme 5, product **31**) or with photoswitchable oligomeric scaffolds bearing terminal alkyne groups (Scheme 5).

**Scheme 5:**
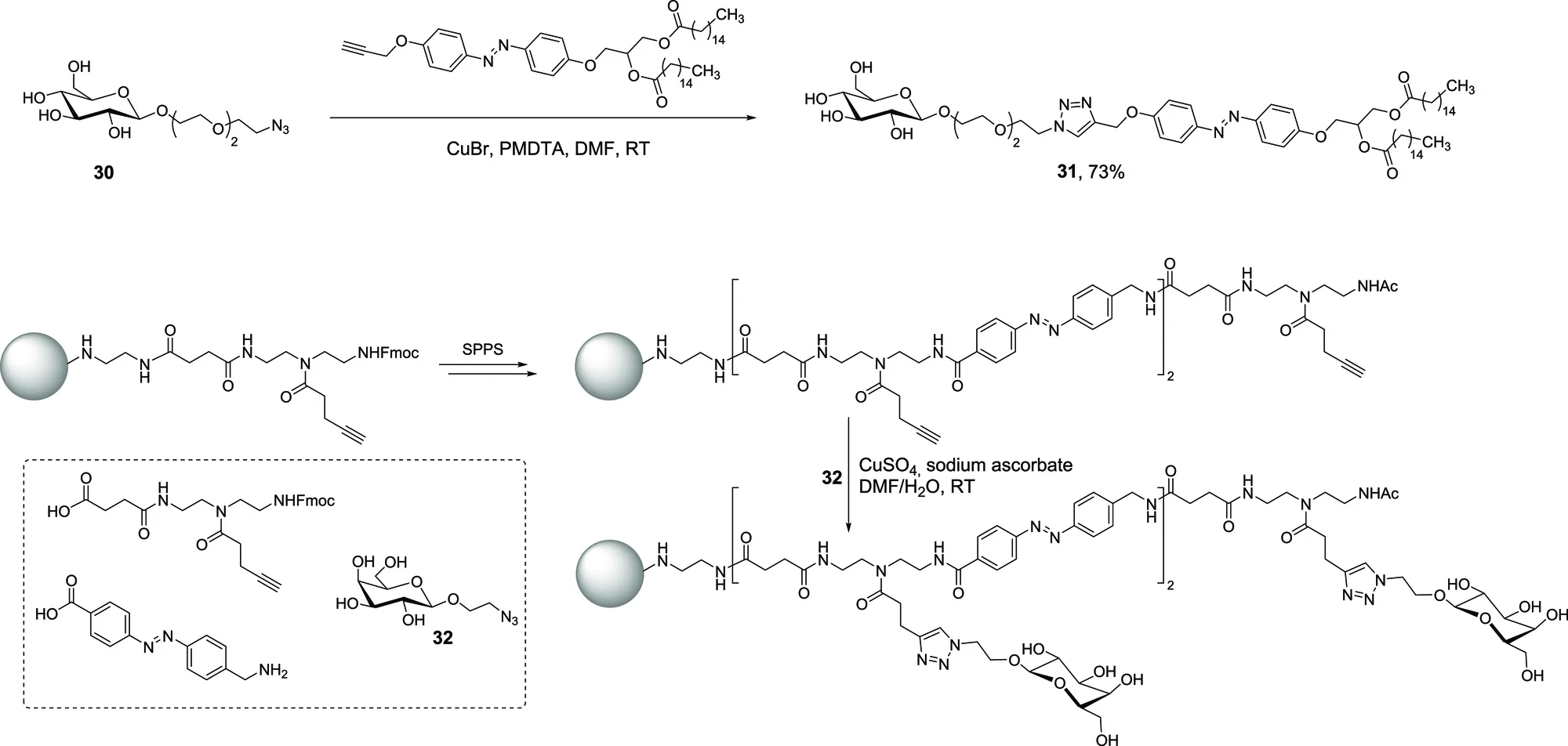
Synthesis of Photoswitchable Glycolipid and Glycooligomer Mimetics Using a Linker-Mediated Design^a^

a PMDTA: *N*,*N*,*N*′,*N*″,*N*″-pentamethyldiethylenetriamine; SPPS: solid phase peptide synthesis.

In addition, ABGs derived from galactosylceramide as CD1d–NKT ligands (Scheme 6, **35** and **36**) were synthesized via amide coupling between a galactosyl sphingosine derivative (**33** or **34**) and azobenzene bearing a terminal carboxylic acid function (Scheme 6).^71,74^

**Scheme 6:**
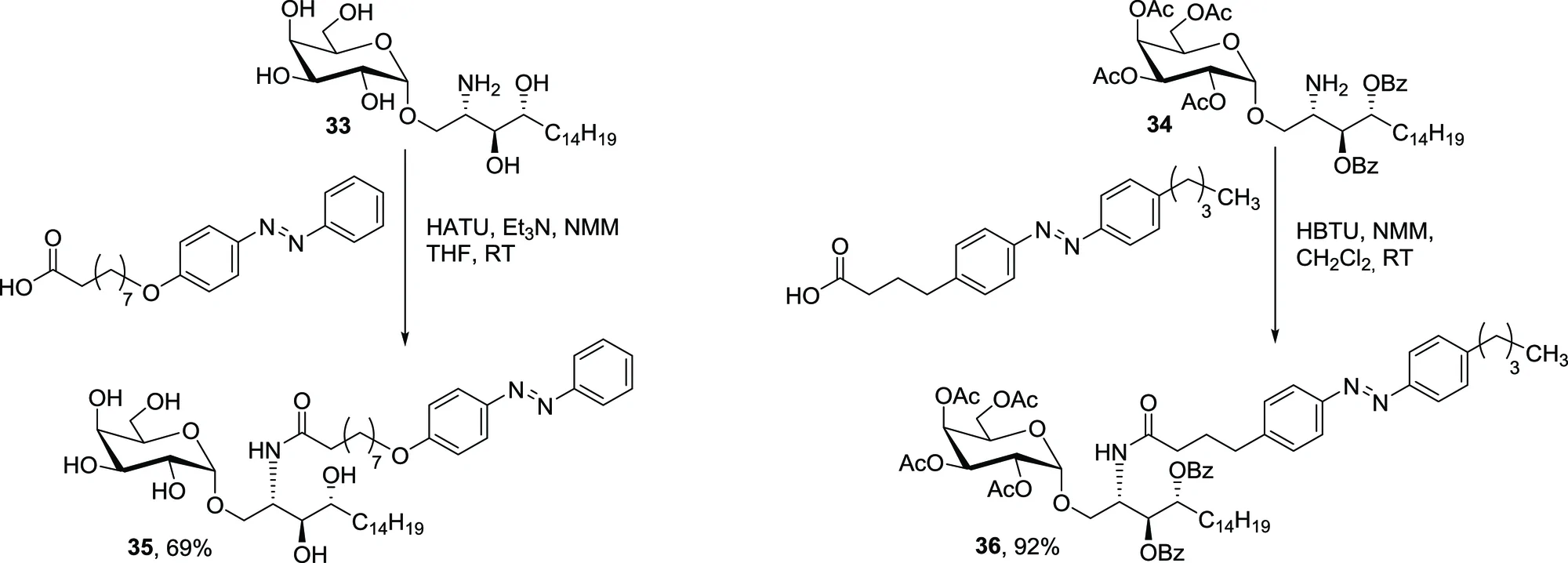
Synthesis of Photoswitchable Galactosylceramides^a^

a HATU: *O*-(7-azabenzotriazol-1-yl)-*N*,*N*,*N′*,*N′*-tetramethyluronium hexafluorophosphate; HBTU: 2-(1*H*-benzotriazol-1-yl)-1,1,3,3-tetramethyluronium hexafluorophosphate; NMM: *N*-methyl-morpholine.

Interestingly, the azobenzene chromophore has also been conjugated to the endocyclic nitrogen of azasugars, which are relevant as photoswitchable glycosidase substrates. Both amide coupling approaches (Scheme 7, products **37** and **38**) and direct thiourea formation have been employed.^72,75^ In the latter case, 1-deoxynojirimycin was reacted with azobenzene isothiocyanate derivatives (Scheme 7, **39**) to afford thiourea-bridged ABGs (**40**) and the corresponding iminothiazolidines (**41**) derived thereof (Scheme 7).

**Scheme 7:**
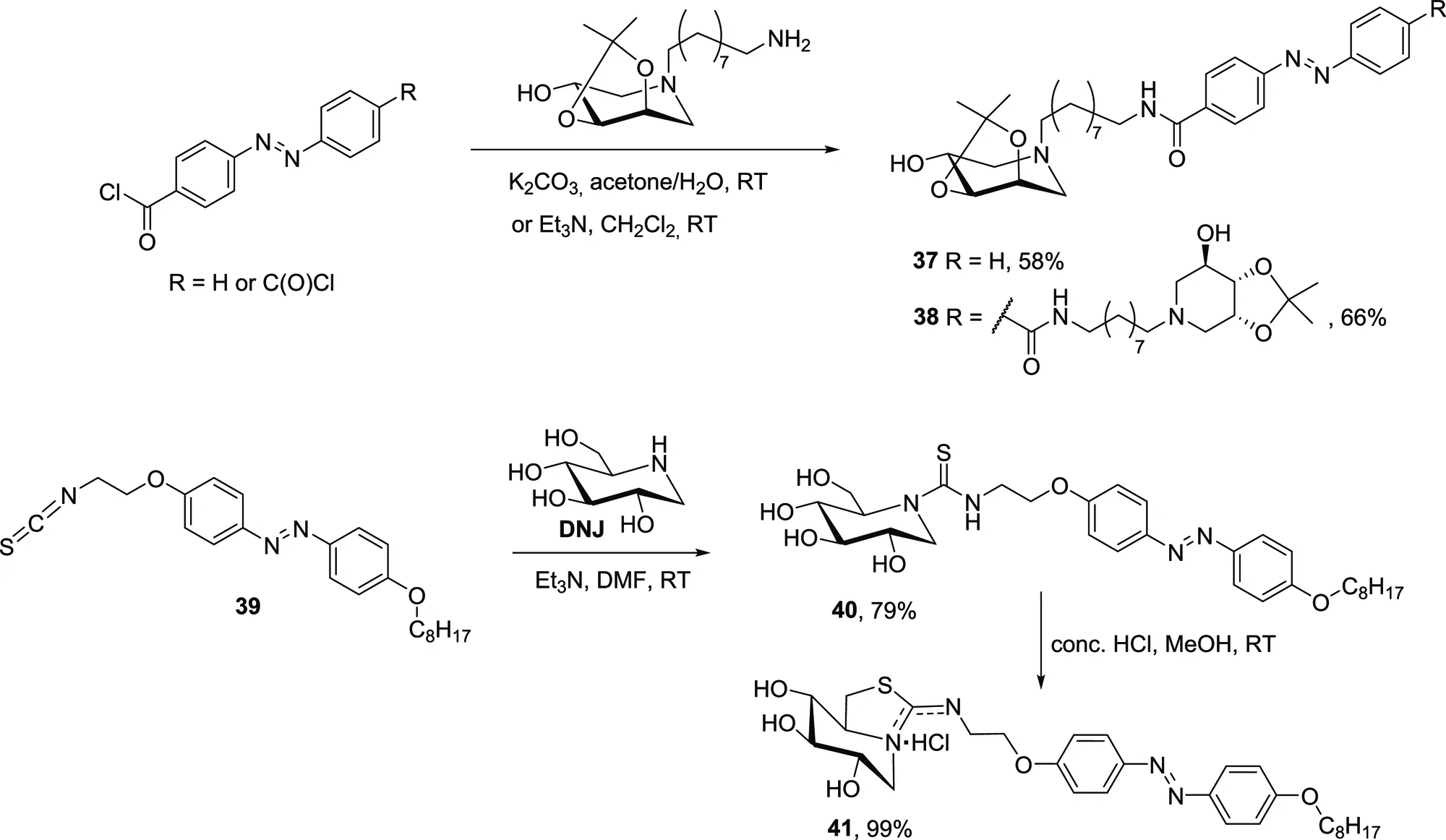
Derivatization of Azasugars Such as 1-Deoxynojirimycin (DNJ) into ABGs

Taken together, the choice between direct and linker-mediated glycan–azobenzene conjugation, and the corresponding use of glycosylation, thiourea formation, triazole formation, amide or thiourea coupling, is governed by whether the primary objective is local epitope reorientation (e.g., in lectins, glycosidases, or CD1d–NKT systems) or global reorganization of glycolipid or self-assembled architectures.

## 6 Azobenzene Glycoconjugates across Structural Scales: Regulation of Biological Recognition

In optoglycomics, photoswitchable glycoconjugates, most prominently azobenzene glycoconjugates (ABGs), are employed to interrogate and regulate biological function with light. As discussed above, optoglycomic studies to date have focused primarily on three classes of carbohydrate-binding proteins, which also define the scope of this Perspective: lectins, glycosidases, and CD1d-NKT. In addition, ABGs have been used for the investigation of membrane models. Accordingly, this chapter is organized around these four areas of application.

Within these biological contexts, ABGs have been implemented in several organizational formats. They can function as discrete soluble ligands, be immobilized on artificial or cellular surfaces, or be incorporated into self-assembled and other supramolecular architectures, including host–guest complexes. These different organizational modes are illustrated in Figure 7. In all cases, light-induced *E*/*Z* isomerization of the azobenzene unit, which acts as a molecular hinge, produces a pronounced conformational change that can be translated into functional control. As highlighted in the following sections, this strategy enables modulation of ligand affinity, multivalence and cooperative effects, and also permits the reversible assembly, disassembly, or structural reorganization of supramolecular constructs. On surfaces, *E*/*Z* isomerization of the azobenzene moiety can reversibly alter the spatial presentation of glycoligands while preserving the overall integrity of the underlying architecture.

**Figure 7:**
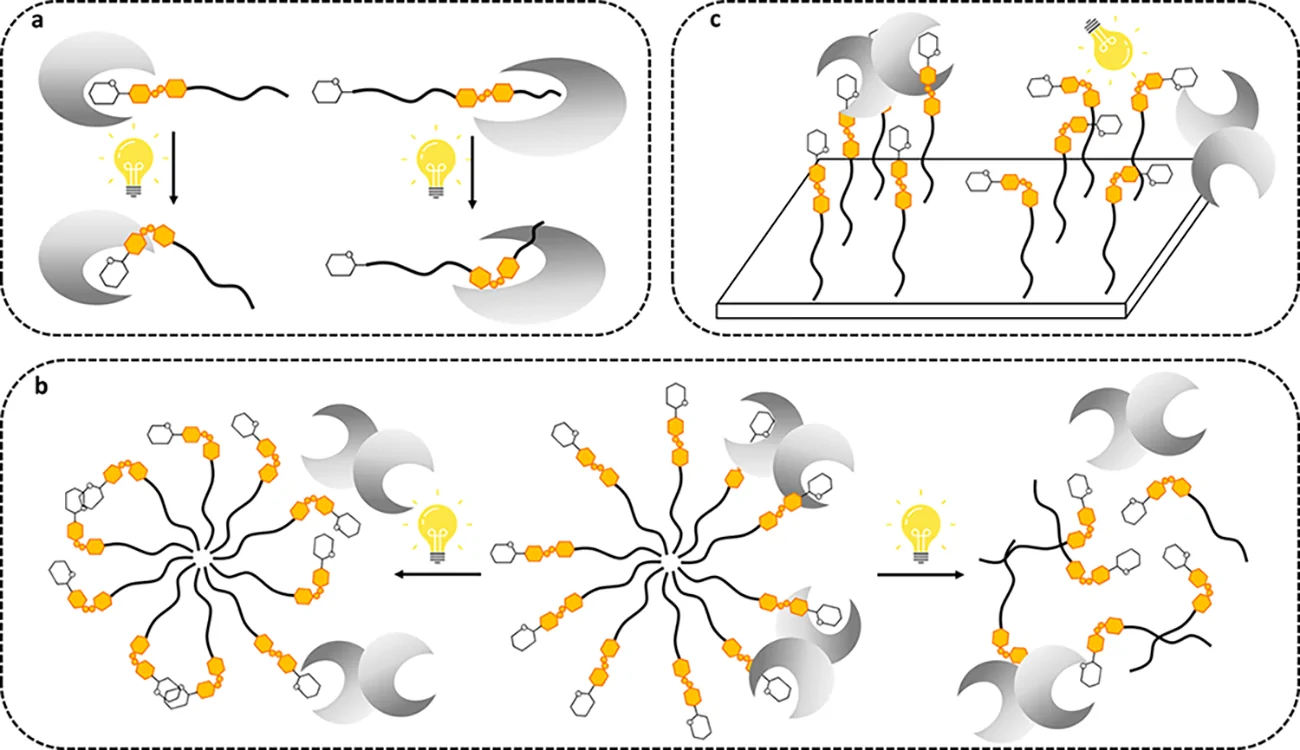
Different organizational modes of ABGs interacting with proteins. (a) Soluble AGBs. (b) ABGs grafted on surfaces such as in self-assembled monolayers on gold. (c) AGBs as part of supramolecular constructs.

### 6.1 Lectins

Understanding the molecular determinants that govern lectin–glycocalyx interactions, such as in microbial adhesion, has long been a central objective in glycobiology. Among bacterial lectins, FimH has emerged as one of the best-studied model systems. Over the past two decades, extensive efforts have been devoted to elucidating the molecular basis of FimH-mediated bacterial adhesion and to developing potent FimH antagonists as antiadhesive therapeutics.^76,77^ To this end, numerous mono- and multivalent glycoconjugates have been synthesized^78^ and evaluated as FimH ligands in a variety of binding and adhesion assays, most notably bacterial adhesion-inhibition assays employing fluorescent *E. coli* cells.^79^ Since 2012, photoswitchable azobenzene glycoconjugates (ABGs) have extended this toolbox by enabling the dynamic regulation of ligand presentation through light-controlled changes in glycoligand orientation and multivalency.^80^

The first photoswitchable monovalent FimH ligand, reported in 2013, consisted of a 1,3-linked mannobioside connected to azobenzene through an α-glycosidic linkage.^58^ Azobenzene mannobioside **42** (Figure 8), prepared by glycosylation of MHAB, exhibited efficient *E*/*Z* photoisomerization and slow thermal relaxation in aqueous solution. Both photoisomers inhibited FimH-mediated bacterial adhesion substantially more potently than the reference ligand methyl α-d-mannoside (MeMan), largely owing to the azobenzene aglycon. The carbohydrate recognition domain (CRD) of FimH is flanked by two tyrosine residues that form a ″tyrosine gate″, enabling favorable π–π interactions with aromatic aglycons and thereby enhancing ligand binding beyond mannose recognition.^81^

**Figure 8:**
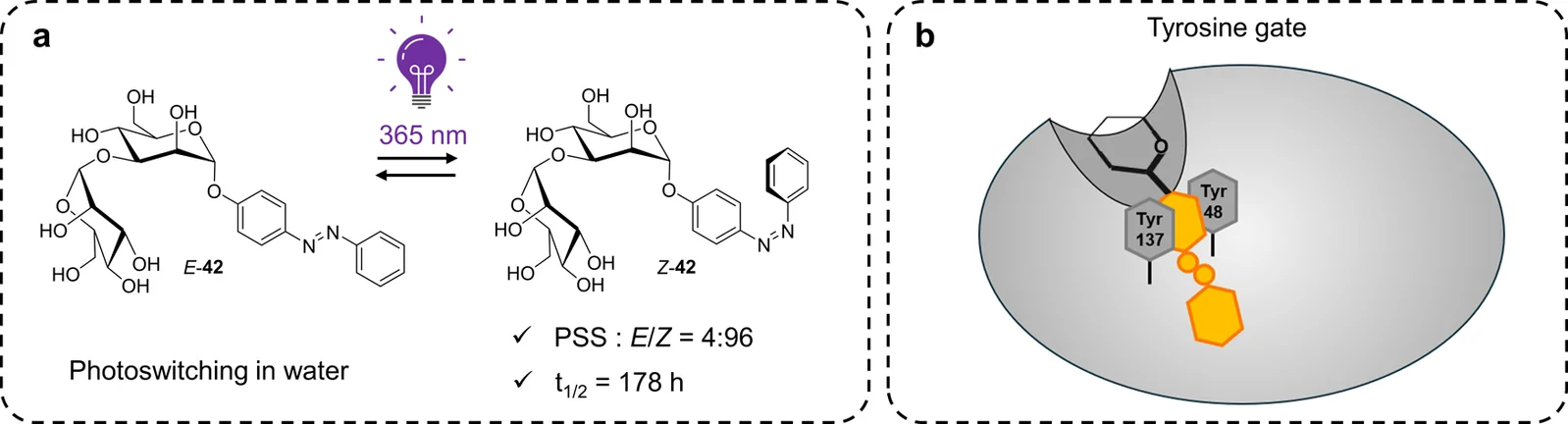
First example of soluble ABG as photoswitchable FimH ligand. (a) Photochromic properties of **42** in water. (b) The tyrosine residues 48 and 137, close to the lectin’s CRD, accommodate aromatic aglycon moieties.

However, only a marginal difference in activity was observed between the two photoisomers of **42**, *E* and *Z*.

In light of these findings, it was hypothesized that the effect of *E*/*Z* isomerization on bacterial lectin recognition would become more pronounced when the glycoligands were immobilized on a surface, where photoisomerization could induce larger changes in ligand presentation. Consequently, the Lindhorst group turned to photoswitchable mannosides, which were displayed on self-assembled monolayers (SAMs) and on the surface of living cells, as discussed below.

At about the same time, the first multivalent azobenzene cluster glycosides bearing three azobenzene glycoside antennas were synthesized and their photochemical properties investigated (Figure 9). Although these compounds were readily accessible in only a few synthetic steps, selective photoaddressing of the individual azobenzene branches was not possible. Moreover, photoconversion to the all-*Z* isomer remained incomplete, likely owing to steric constraints and the close spatial proximity of the azobenzene units within the cluster.^57^

**Figure 9:**
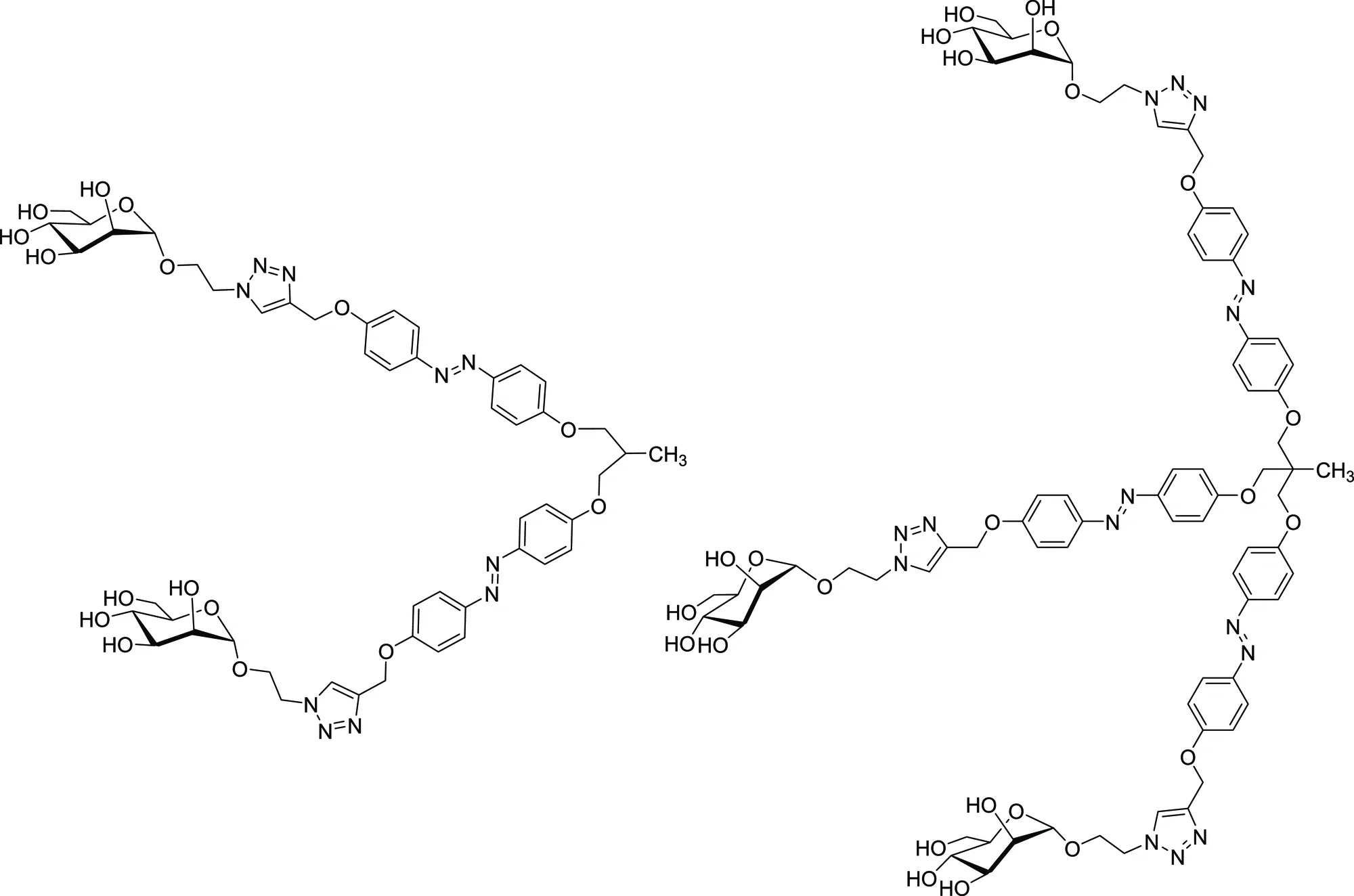
First cluster glycosides bearing two or three photoswitchable AB-based antennas.

More recently, the Lindhorst group advanced the optoglycomics concept by introducing a new azobenzene glycoconjugate (ABG) scaffold containing two spectrally distinct azobenzene photoswitches, thereby enabling orthogonal photoswitching (Figure 10).^5,82^ Compound **43** is a heterobivalent glycocluster comprising two independently addressable glycan antennas, an α-mannoside and a β-glucoside, attached to a central 3,6-branched mannoside scaffold. This molecular architecture is particularly relevant for investigating carbohydrate–protein interactions because the 3,6-branched mannose motif is a characteristic structural element of naturally occurring high-mannose *N*-glycans. To construct the two photoresponsive antennas, conventional *O*-glycosylation was combined with thioarylation, introducing a parent azobenzene unit together with a red-shifted tetra-*ortho*-fluoroazobenzene (ABF_4_). For comparison, the corresponding homobivalent dimannosyl cluster **44**, as well as three monovalent analogues **45**, **46**, and **47** (Figure 10) representing the individual antennas, were synthesized using the same strategy.

**Figure 10:**
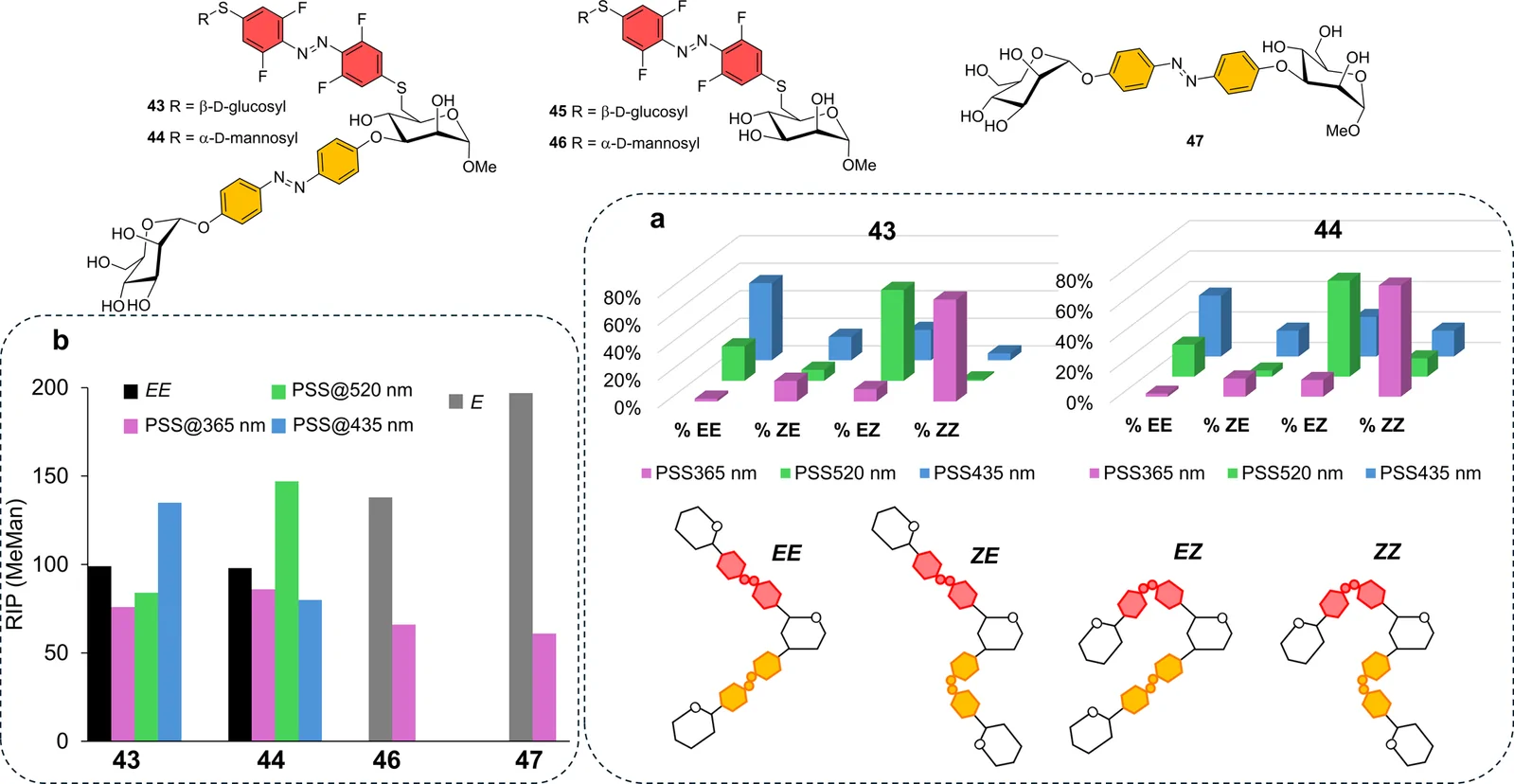
Heterobivalent cluster glycosides bearing two orthogonally photoswitchable antennas. (a) Orthogonal photoswitching of compounds **43** and **44** upon irradiation with UV-, green- or blue-light: four isomers coexist in each corresponding PSS. (b) Comparison of RIP values measured with **43** and **44**, plus the single antennas derivatives **46** and **47**, toward adhesion inhibition of type-1 fimbriae *E. coli* bacteria. RIP: relative inhibitory potency.

Photochemical characterization demonstrated efficient wavelength-selective photoisomerization of the heterobivalent glycocluster (Figure 10a). Of the four possible configurational states (*EE*, *EZ*, *ZE*, and *ZZ*), irradiation at 365, 435, and 520 nm generated three distinct photostationary states enriched predominantly in the *ZZ*, *EE*, and *EZ* configurations, respectively. Importantly, the *E/Z* ratios obtained for each photoswitch within the heterobivalent scaffold closely matched those measured for the corresponding monovalent analogues, demonstrating that the two azobenzene units operate largely independently, a prerequisite for true orthogonal photoswitching. The metastable *Z* isomers also exhibited sufficiently slow thermal relaxation for biological experiments, with the tetra-*ortho*-fluoroazobenzene unit displaying the highest thermal stability, as expected.^49^

Standard bacterial adhesion-inhibition assays revealed that binding of the bivalent glycoclusters to type 1-fimbriated *E. coli* depends on the photostationary state (Figure 10b). The monovalent mannose-containing antennas exhibited more pronounced differences between the *E* and *Z* states than the heterobivalent glycoconjugates, reflecting the higher *Z*-isomer content achieved in their respective photostationary states. Molecular docking and molecular dynamics simulations helped rationalize these observations. Whereas the computational results for the monovalent ABGs closely mirrored the experimental data, modeling of the heterobivalent scaffold showed that the terminal α-mannoside consistently occupies the FimH carbohydrate recognition domain (CRD), while the second antenna establishes variable peripheral interactions. These findings reveal a complex and only partially intuitive structure–activity relationship, in which multivalency and conformational flexibility obscure a direct correlation between photostate and inhibitory potency. Nevertheless, the study demonstrates that light-controlled reorientation of glycoligands provides a versatile means to modulate bacterial adhesion.

Another representative class of lectin-targeting ABGs comprises ligands for the *P. aeruginosa* lectins LecA and LecB.^83^ These tetrameric lectins play pivotal roles in bacterial adhesion, biofilm formation, and virulence, making them attractive targets for antiadhesive therapeutic strategies.^84^ The design of photoswitchable LecA/LecB ligands has benefited from an extensive body of work on mono- and multivalent glycoconjugate inhibitors.^85^

Xie and co-workers recently reported a series of monovalent galactose-based ABGs targeting LecA.^55^ *O*-Linked azobenzene galactosides were prepared by DMC-mediated glycosylation of *para*- and *meta*-substituted dihydroxyazobenzenes (DHABs) in water, whereas the corresponding azobenzene thiogalactosides, together with an *ortho*-substituted analogue, were synthesized via Buchwald–Hartwig–Migita cross-coupling. To improve water solubility, all compounds were functionalized with a terminal ammonium group. Similar to the FimH-targeting ABGs described above, compounds **48**–**50** (Figure 11) displayed excellent photoswitching behavior and sufficiently slow thermal relaxation for binding studies under aqueous conditions.

**Figure 11:**
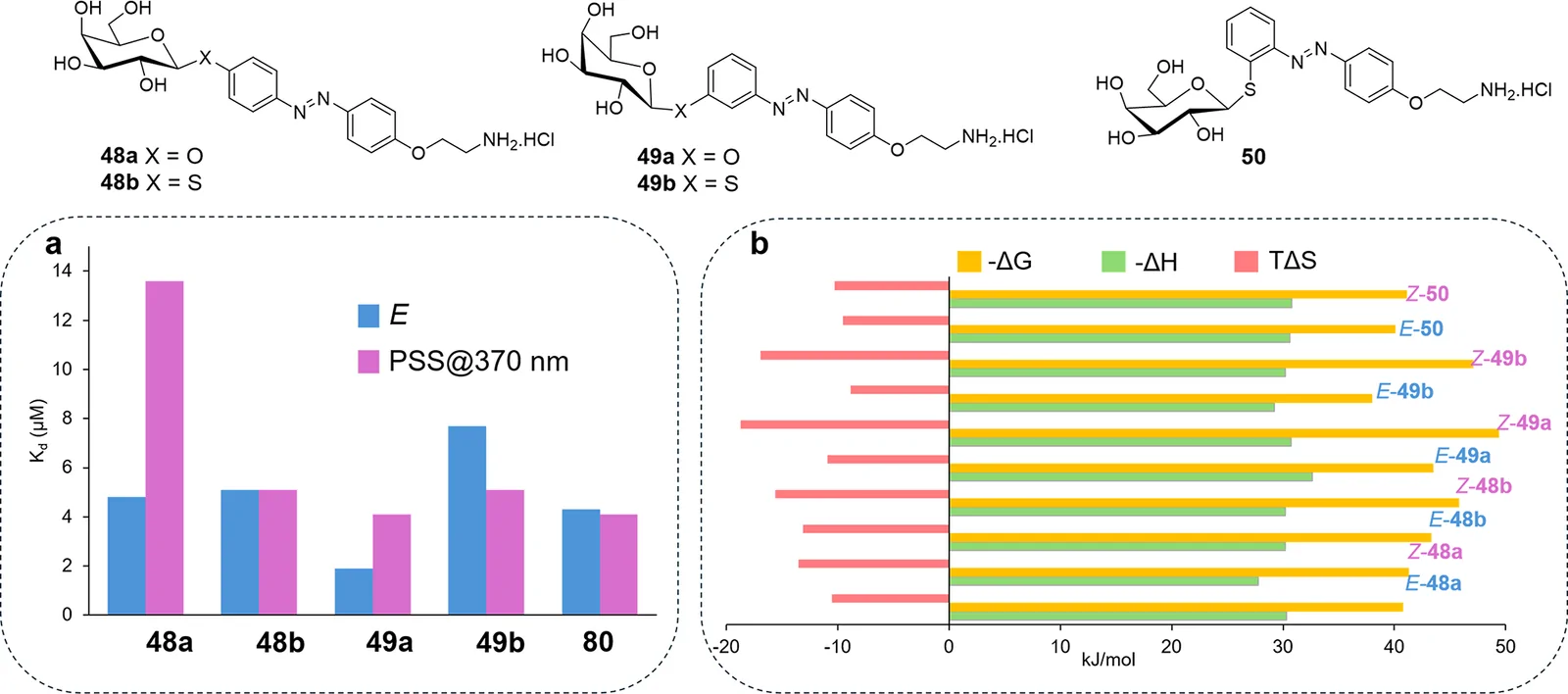
Evaluation of *para*-, *meta*- and *ortho*-substituted AB *O*-glycosides and AB thioglycosides toward LecA binding. (a) Comparison of dissociation contant (*K*_d_) values as measured by ITC. (b) Comparison of thermodynamic parameters of Lec A binding, deduced from the ITC measuremements. ITC: isothermal titration calorimetry.

Binding to LecA was investigated by isothermal titration calorimetry (ITC), allowing determination of both binding affinities and the corresponding thermodynamic parameters for the pure *E* isomers and *Z*-enriched photostationary-state mixtures. All ABGs formed 1:1 complexes with LecA. Notably, the *para*-substituted galactosides exhibited a two- to 3-fold higher affinity in the *E* configuration than in the corresponding *Z*-enriched mixtures, whereas the *meta*-substituted analogues showed little dependence on the photostate (Figure 11a). Thermodynamic analysis revealed that binding of the *Z* isomers was associated with more favorable enthalpic contributions but also with a substantially larger entropic penalty. As a result, the overall binding free energy remained more favorable for the *E* isomers, highlighting the subtle balance between enthalpic and entropic effects that governs photoswitch-dependent lectin recognition (Figure 11b).

Isothermal titration calorimetry (ITC) was likewise employed by Parsini and co-workers to investigate the interaction of a photoswitchable l-fucoside derivative with LecB. Consistent with the observations for the LecA-targeting ABGs, the UV-irradiated sample (365 nm), enriched in the *Z* isomer of **21** (see Scheme 3 for structure), displayed an approximately 2-fold lower binding affinity than the corresponding *E* isomer. Analysis of the thermodynamic parameters again revealed enthalpy–entropy compensation, with the more favorable overall binding free energy observed for the *E* configuration.^65^

Multivalent ABGs have also been developed to modulate LecA recognition. Hartmann and co-workers incorporated azobenzene hinges into sequence-defined oligoamides bearing pendant β-d-galactosides introduced by CuAAC chemistry.^73^ The resulting precision glycooligomers were designed to match the spacing between adjacent carbohydrate recognition domains (CRDs) in the tetrameric LecA assembly (Figure 12). In competitive SPR assays, the *E* isomer of the digalactosyl oligomer **51** and the *E*,*E* isomer of the trigalactosyl analogue **52** inhibited LecA binding with low-micromolar IC_50_ values comparable to those of nonphotoswitchable reference compounds. Photoisomerization, however, significantly affected activity only for **51**, as contraction of the *Z* isomer reduced the galactose–galactose distance below that required to bridge adjacent LecA CRDs. In contrast, the doubly switched *Z*,*Z* isomer of **52** retained sufficient spacing for simultaneous binding to neighboring sites, consistent with molecular modeling.

**Figure 12:**
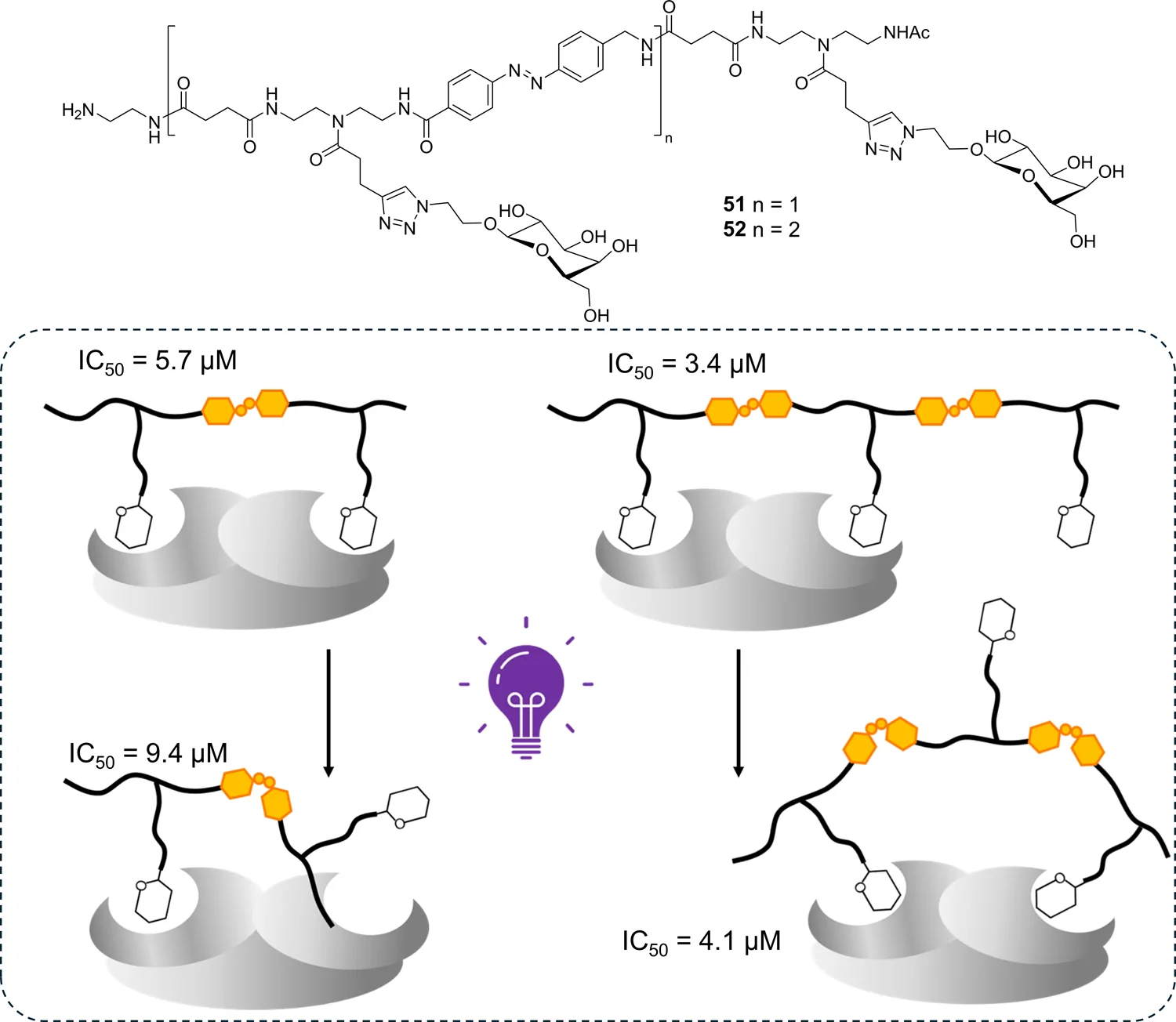
Photoswitchable oligomers based on d-galactose for targeting LecA. The given IC_50_ values were obtained by competitive adhesion inhibition assays.

The authors also immobilized the glycooligomers on SPR sensor surfaces to investigate surface-confined lectin recognition. Under these conditions, photoisomerization significantly affected binding only for the trigalactosyl oligomer, whereas the digalactosyl derivative displayed the highest overall affinity. The modest photoswitching response was attributed to restricted accessibility of one galactose residue located too close to the surface to participate effectively in LecA binding.

#### 6.1.1 Photoswitchable Supramolecular Glycoassemblies

Beyond their use as discrete ligands, ABGs can serve as building blocks of self-assembled supramolecular architectures. Incorporation into such nano- and microscale assemblies adds an additional level of functional complexity, as light can be used not only to modulate ligand conformation but also to influence multivalent glycan presentation and, potentially, the structure and properties of the assembly itself. The following examples illustrate how these concepts have been explored to control lectin recognition and related biological functions.

In 2016, Wilkinson and co-workers reported the first use of photoswitchable glycolipid surfactants to control bacterial growth and biofilm formation, including those of *P. aeruginosa*, with light.^86^ Subsequently, the same group developed photoswitchable glycodendrimers that spontaneously assemble into micelles displaying α-l-fucosyl, α-d-mannosyl, or β-d-galactosyl residues in a multivalent fashion.^67^ Light and neutron scattering experiments revealed the formation of relatively monodisperse cylindrical micelles with radii of approximately 3–4 nm and lengths ranging from 10 to 100 nm (Figure 13).

**Figure 13:**
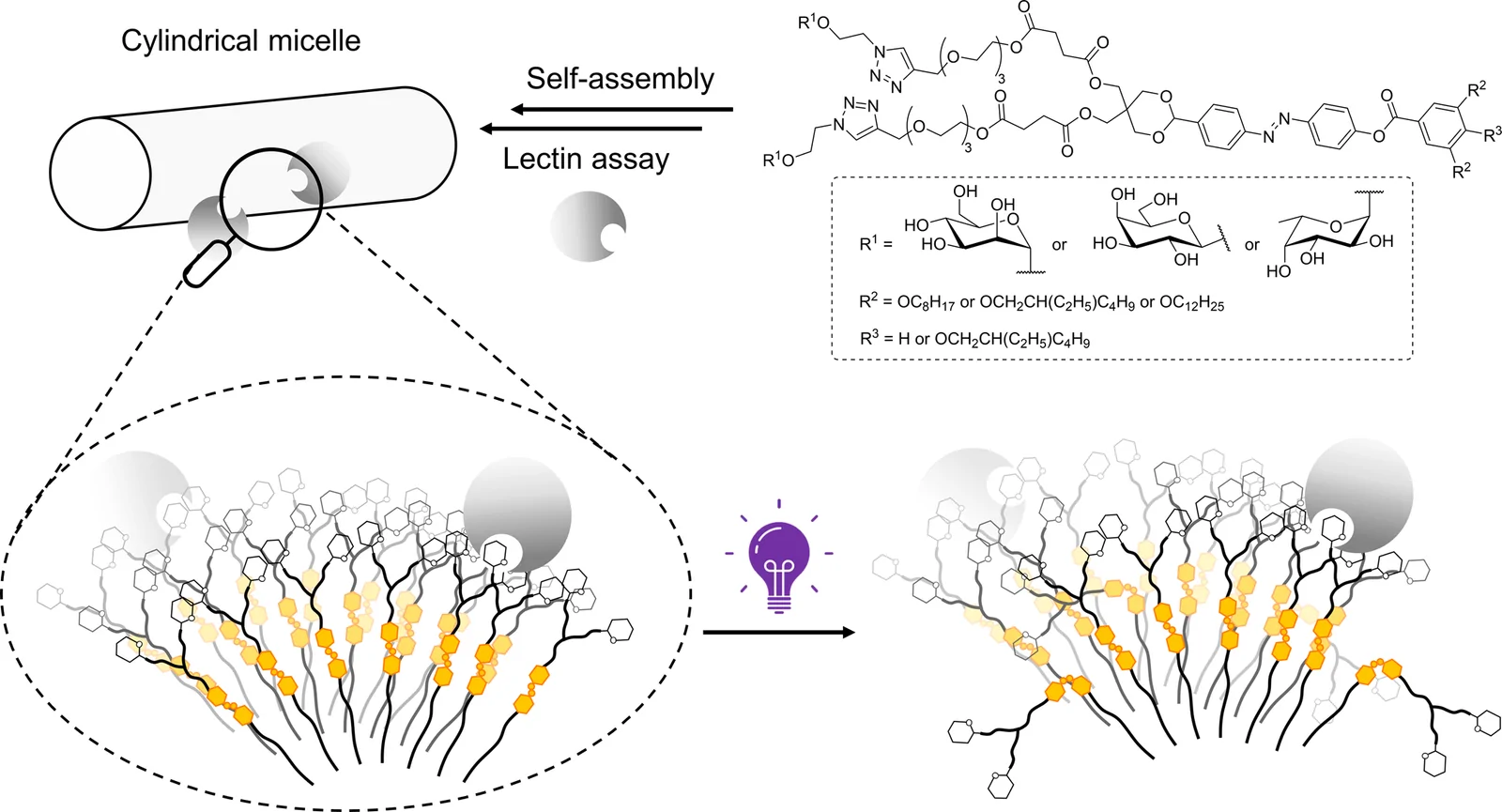
Photoswitchable Janus glycodendrimers targeting LecA or LecB. Cylindrical micelles characterized by DLS and SANS techniques display the glycoside ligands in tightly packed arrays, thus impairing proper photoswitching. DLS: dynamic light scattering; SANS: small-angle neutron scattering.

The glycomicelles were evaluated as inhibitors of the lectins LecA (galactose-specific) and LecB (fucose- and mannose-specific), exhibiting moderate to high inhibitory potencies. Comparison with the corresponding methyl glycosides showed little evidence for a multivalence effect upon lectin binding. Furthermore, UV irradiation neither induced measurable changes in micelle size or morphology nor altered their lectin-binding properties. These observations are consistent with the photochemical behavior of the assemblies, which exhibited only limited conversion to the *Z* isomer upon irradiation in aqueous solution. The reduced photoswitching efficiency is most likely a consequence of the dense packing of the azobenzene units within the micellar core, which restricts the molecular motions required for efficient *E*/*Z* isomerization.

More recently, photoswitchable neoglycolipids comprising a mannosyl headgroup and an amphiphilic alkyl chain have been developed.^87^ In particular, compound **53** forms supramolecular gels in water/ethanol mixtures at remarkably low concentrations, with gelation occurring at weight fractions as low as 0.005% (Figure 14). Electron microscopy revealed networks of interwoven tubular aggregates with lengths on the micrometer scale and uniform diameters and wall thicknesses of approximately 30 and 10 nm, respectively. Small-angle X-ray scattering (SAXS) further demonstrated that the tube walls consist of a single lipid bilayer, consistent with the mannosyl residues being exposed at the outer surface of the nanotubes.

**Figure 14:**
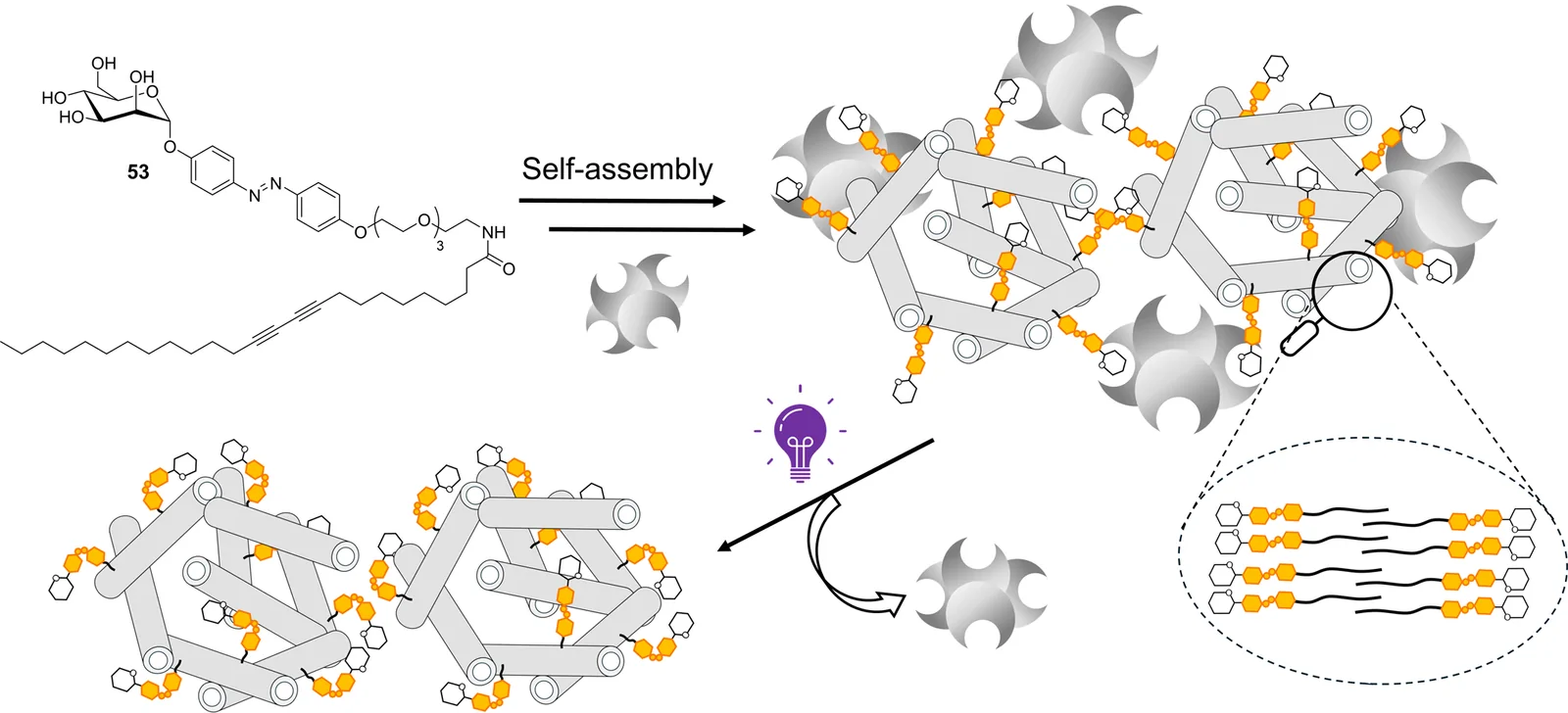
D-Mannose-based photoswitchable neoglycolipid; compound **53** self-assembles in the form of interlocked nanotubules, providing a photostable gel. Catch and release of ConA is due to spatial reorientation of the mannosyl ligands upon *E* → *Z* photoisomerization of the AB units.

Notably, these gels retain excellent photoswitching properties under both UV and visible light. At low ABG concentrations, *E* → *Z* photoisomerization induces a reversible gel-to-sol transition, a feature that was exploited to achieve light-triggered release of rhodamine B, illustrating the potential of this platform for controlled drug delivery. The authors further demonstrated reversible light-controlled capture and release of the lectin ConA. At a higher neoglycolipid content (0.5 wt %), efficient photoisomerization could be achieved while preserving the gel state, such that irradiation altered only the spatial presentation of the mannosyl ligands without disrupting the supramolecular architecture. In the *E* state, ConA binds to and aggregates the glyconanotubes, whereas photoisomerization to the *Z* state promotes lectin release, presumably because the altered orientation of the surface-displayed mannose residues weakens multivalent binding.

Host–guest chemistry provides another attractive strategy for constructing higher-order optoglycomic systems. In particular, azobenzene derivatives readily form inclusion complexes with water-soluble macrocycles such as cyclodextrins and cucurbiturils, enabling light-responsive supramolecular assembly through reversible *E*/*Z* photoisomerization.^88^ This approach was pioneered by Ravoo and co-workers, who exploited the self-assembly of amphiphilic cyclodextrins into vesicles displaying ABGs at their surface through inclusion of the azobenzene moiety within the cyclodextrin cavity.^89^ The resulting glycovesicles served as light-responsive lectin receptors, capturing lectins in the assembled state and releasing them upon irradiation. Photoisomerization to the *Z* configuration weakened the host–guest interaction, leading to depletion of the ABGs from the vesicle surface and concomitant lectin release.

In 2021, Bi and co-workers expanded this strategy by designing a hierarchical supramolecular system combining metal coordination with host–guest recognition (Figure 15a).^90^ In a first step, coordination of a methyl viologen–pyridine ligand (**54**) with a platinum complex (**55**) yielded either rhomboidal or hexagonal metallacycles. Subsequent complexation with cucurbit[8]­uril (**CB­[8]**) and a simple azobenzene galactoside (**56**) afforded glycometallacycles displaying either two or three galactosyl ligands. Depending on their geometry, these building blocks further self-assembled in water into multilamellar vesicles with diameters of approximately 50 nm or into rod-like microstructures with diameters of about 100 nm.

**Figure 15:**
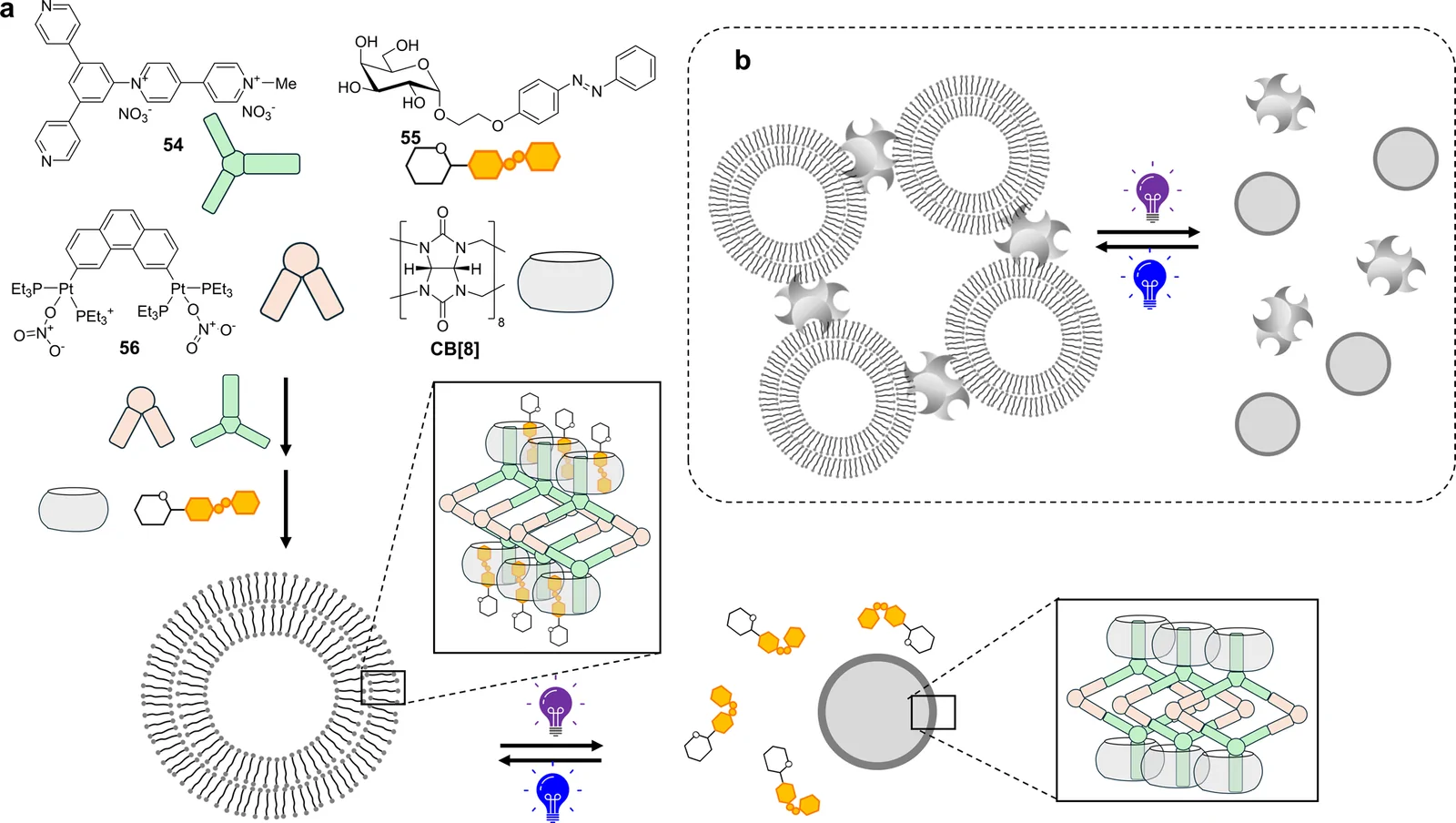
Self-assembling photoswitchable supramolecular constructs exposing galactosyl ligands. (a) Step-by-step hierarchical assembly of multilamellar vesicles and their reversible light-induced destruction/reforming. (b) Application to the reversible light-controlled catch and release of PNA.

Upon UV irradiation, photoisomerization of the azobenzene unit disrupted the CB[8]-mediated host–guest interaction, triggering disassembly of the supramolecular assemblies into smaller nanostructures. Taking advantage of the high affinity of peanut agglutinin (PNA) for multivalent galactosides, the authors demonstrated that the assembled vesicles and rods efficiently induced lectin aggregation, as monitored by turbidity measurements. Irradiation released the bound lectin by abolishing the multivalence effect through disassembly of the supramolecular scaffold. As in the earlier cyclodextrin-based system, the process was fully reversible, allowing repeated cycles of light-controlled lectin capture and release (Figure 15b).

Beyond ConA and FimH, D-mannosides are also recognized by dendritic cells (DCs), which function as key mediators between the innate and adaptive immune systems via lectins such as DC-SIGN.^91^ Cancer cells have likewise been reported to overexpress mannose-binding lectins at their surface.^92^ In this context, a photoswitchable mannosylated oligomer incorporating azobenzene units within its backbone was designed to modulate recognition by both cancer cells and DCs, with the aim of promoting cancer cell death while enhancing DC maturation.^68^

Compound **57** self-assembles in aqueous media into microscale helical fibers, as revealed by transmission electron microscopy (TEM). Upon UV-induced *E* → *Z* photoisomerization, these fibers convert into small spherical micelles. Although visible light irradiation does not fully regenerate the original fibrous morphology, the system undergoes a fully reversible and repeatable fiber–micelle transition over multiple UV/visible light cycles (Figure 16a).

**Figure 16:**
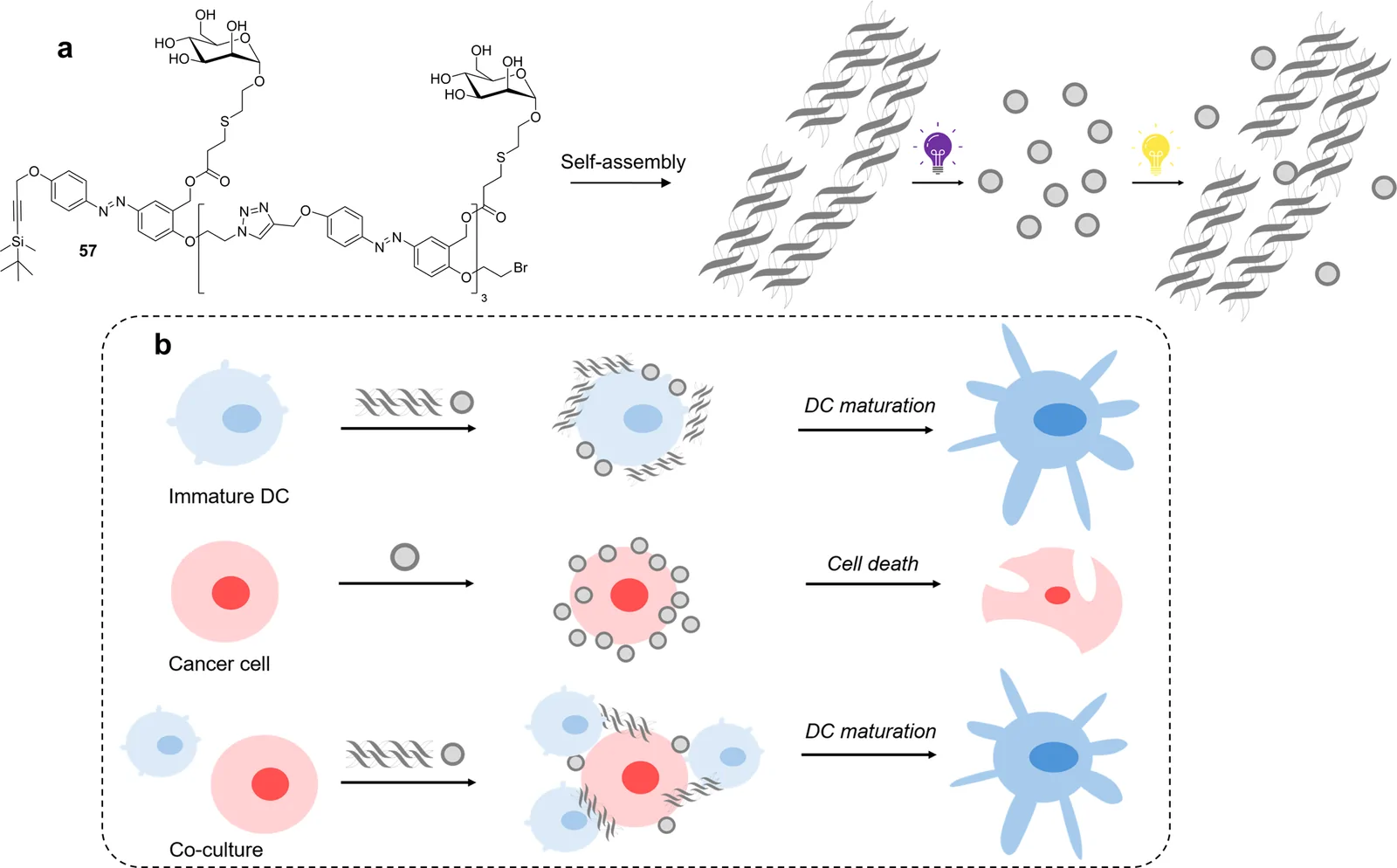
d-Mannose-grafted photoswitchable oligomers for modulating DC maturation and induce cancer cells death. (a) Self-assembly of **57** to helical fibers that undergo transition to smaller micelles upon UV-light exposure; visible-light irradiation leads to a mixture of fibers and micelles. (b) Use of oligomers/micelles mixtures to promote DC maturation or of micelles to induce cancer cells death. DC: dendritic cells.

Biological evaluation was performed using cancer cells and DCs, both in monoculture and in coculture systems, in the presence of either fiber-dominated assemblies (corresponding to the *E* photostationary state) or micelle-rich mixtures (corresponding to the UV-induced *Z* photostationary state). The results showed that the micellar form of **57** is more effective at reducing cancer cell viability, whereas fiber-rich assemblies more efficiently promote DC maturation, particularly in coculture conditions. This enhanced immune activation likely arises from a combination of direct interactions between the self-assembled glycostructures and DCs, as well as from assembly mediated cell–cell contact between DCs and cancer cells (Figure 16b).

The studies discussed in this paragraph highlight that the scope of ABGs extends well beyond soluble photoswitchable lectin ligands. Their integration into organized soft-matter systems enables spatiotemporal control over both structure and biological function. Through reversible modulation of multivalent presentation, assembly size and morphology, membrane organization, or structural integrity, ABGs provide a versatile platform for regulating lectin binding, microbial inhibition, cargo release, and cell–cell recognition.

#### 6.1.2 Photoswitchable Glyco-SAMs: Optical Control of Glycan Presentation at Interfaces

Studies of carbohydrate-mediated recognition have repeatedly demonstrated that biological function is governed not only by the stereochemical identity of individual glycans but also by their spatial organization in multivalent and supramolecular environments.^93,94^ The density, orientation, and presentation of glycans critically influence processes such as bacterial adhesion to glycosylated surfaces and the formation of glycosynaptic microdomains by glycosphingolipids.^95,96^ Despite these insights, suitable glycomimetic model systems that allowed the isolated investigation of glycan orientation with molecular precision remained unavailable for many years.

This challenge was addressed by the development of photoswitchable glyco-surfaces based on azobenzene glycosides. Reversible *E*/*Z* photoisomerization of the azobenzene scaffold enables light-induced modulation of the orientation of surface-bound glycans without altering their chemical identity, thereby providing a bottom-up strategy to investigate the role of glycan presentation in carbohydrate recognition.

To organize the photoswitchable glycoligands on a surface, self-assembled monolayers (SAMs) on gold proved particularly well suited. The azobenzene glycosides were equipped with an ω-thiol linker fulfilling three essential functions: (i) the terminal thiol anchors the molecule to the gold surface through formation of a strong Au–S bond; (ii) an alkyl spacer promotes self-assembly through intermolecular hydrophobic interactions; and (iii) an oligo­(ethylene glycol) (OEG) segment suppresses nonspecific protein adsorption, thereby imparting the bioinert character required for biological recognition studies. The glycosidically bound mannoside serves as specific ligand for the bacterial FimH (Figure 17a).^54^

**Figure 17:**
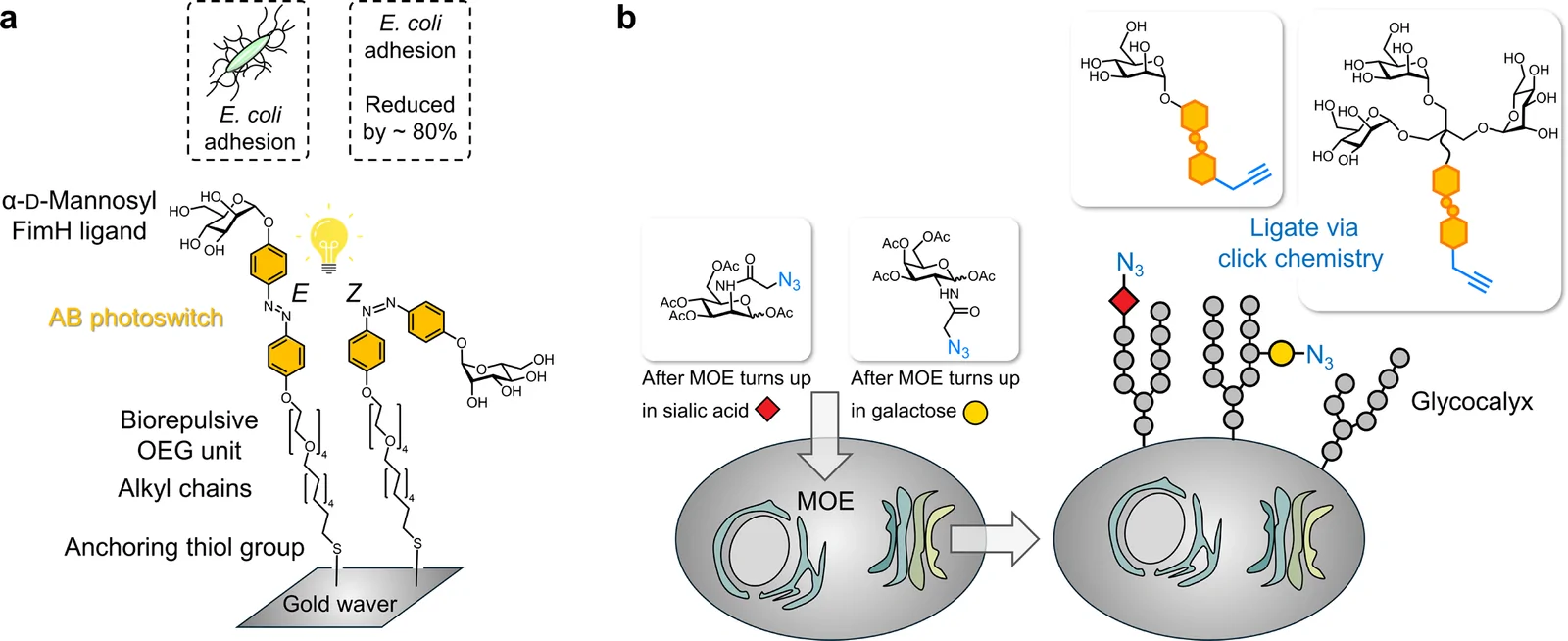
Optoglycomics in surfaces: (a) Photoswitchable glyco-SAMs (self-assembled monolayers) can be reversibly isomerized between an *E* and a *Z* state. In the *Z* state, the orientation of the mannose ligand relative to the surface is altered and consequently, adhesion of *E. coli* is reduced by ∼80%. (b) Metabolic oligosaccharide engineering (MOE) can be employed to label the glycocalyx of cells with unnatural azido functions. These can be addressed in biorthogonal CuAAC click chemistry with various propargylated ABGs.

The target molecules were readily synthesized from 4,4′-dihydroxyazobenzene. Installation of the surface-anchoring linker by a Mitsunobu reaction was facilitated by the use of polymer-supported triphenylphosphine, which greatly simplified purification by avoiding contamination with triphenylphosphine oxide.

Efficient photoisomerization of the glyco-SAMs on gold was confirmed by infrared reflection–absorption spectroscopy (IRRAS). Photoswitching of such glyco-SAMs enabled optical regulation of carbohydrate-mediated adhesion of *E. coli* cells, mediated by the bacterial lectin FimH. Switching between the *E* and *Z* states reduced carbohydrate-specific bacterial adhesion by up to 80%, demonstrating that glycan orientation alone can profoundly influence biological recognition.

Subsequent studies have further optimized the photoswitching performance of glyco-SAMs by modifying the molecular architecture of the photoswitchable backbone.^97^ Although these advances have improved switching efficiency, considerable opportunities remain for the rational optimization of photoswitchable glycointerfaces with respect to switching amplitude, fatigue resistance, and biological function.

These studies provided one of the earliest demonstrations that light can be used to reversibly manipulate glycan presentation at biointerfaces and thus established a conceptual foundation for the emerging field of optoglycomics.

Remarkably, the same concept was subsequently translated from artificial glycointerfaces to the glycocalyx of living human microvascular endothelial cells (HMEC) by combining metabolic oligosaccharide engineering (MOE) with bioorthogonal conjugation chemistry.^98^ In MOE, chemically modified monosaccharides are metabolically incorporated into cellular glycans through the endogenous glycan biosynthetic machinery, thereby introducing bioorthogonal functional groups into defined regions of the glycocalyx.^99^ Azido-functionalized monosaccharides have proven particularly valuable for this purpose, as they enable the selective attachment of alkyne-functionalized probes by click chemistry (Figure 17b).

Photoswitchable azobenzene mannosides carrying a terminal alkyne group could therefore be conjugated to azido-labeled glycans by Cu­(I)-catalyzed azide–alkyne cycloaddition (CuAAC) and subsequently mannose-specific bacterial adhesion to the engineered cell surface was determined. Depending on the metabolically incorporated sugar precursor, the photoswitch could be positioned at different locations within the glycocalyx. Incorporation of *N*-azidoacetylmannosamine (ManNAz) resulted in labeling of terminal sialic acid residues, whereas *N*-azidoacetylgalactosamine (GalNAz) primarily targeted mucin-type *O*-glycoproteins, placing the photoswitch within more internal regions of the glycocalyx.

Real-time fluorescence microscopy revealed that photoswitching of the cell-surface glycans enabled reversible optical control of bacterial adhesion. When ABGs were displayed on terminal sialylated glycans following ManNAz incorporation, reversible *E*/*Z* isomerization significantly modulated the adhesion of GFP-expressing *E. coli*. In contrast, conjugation of the same photoswitchable ligands to mucin-type glycoproteins following GalNAz incorporation increased bacterial adhesion but largely abolished photoswitch-dependent regulation.

This striking difference highlights the importance of the local glycocalyx architecture for optoglycomic regulation. The bacterial lectin FimH recognizes terminal mannosides presented on the host cell surface. When the photoswitchable mannoside is located at the nonreducing terminus of a glycan, photoisomerization produces a pronounced change in ligand accessibility, enabling bacterial adhesion to be reversibly regulated with light. In contrast, positioning the photoswitchable ligand within the densely glycosylated mucin layer largely abolished optical control. These findings demonstrate that successful optoglycomic modulation depends not only on the photoswitch itself but also on its precise spatial positioning within the three-dimensional glycocalyx.

Importantly, this concept is not limited to monovalent ligands but can be extended to more complex molecular architectures. Mannoside clusters were also conjugated to the surface of human microvascular endothelial cells, again enabling reversible bacterial adhesion by optoglycomic regulation of ligand presentation (Figure 17b).^100^

### 6.2 Glycosidases

Glycosidases represent important targets for ABGs, and the development of photoswitchable inhibitors has expanded considerably over the past decade. As noted above, the dysregulation of glycosidases is associated with diseases such as diabetes and lysosomal storage disorders (LSDs). Beyond the concept of light-controlled on/off inhibition, glycosidases present an additional challenge in the context of LSDs, where therapeutic intervention relies on pharmacological chaperones (PCs).^18^

A pharmacological chaperone (PC) is a compound that binds to a glycosidase and promotes its correct folding, thereby stabilizing the enzyme and facilitating its trafficking to the lysosome. Once the complex reaches the lysosome, the PC is displaced by the natural substrate, which can then be properly processed (Figure 18). Accordingly, an ideal PC must form a sufficiently stable complex with the glycosidase to ensure folding and transport, yet bind more weakly than the natural substrate to allow subsequent release. For this reason, PCs are typically applied at subinhibitory concentrations to achieve maximal therapeutic efficacy.

**Figure 18:**
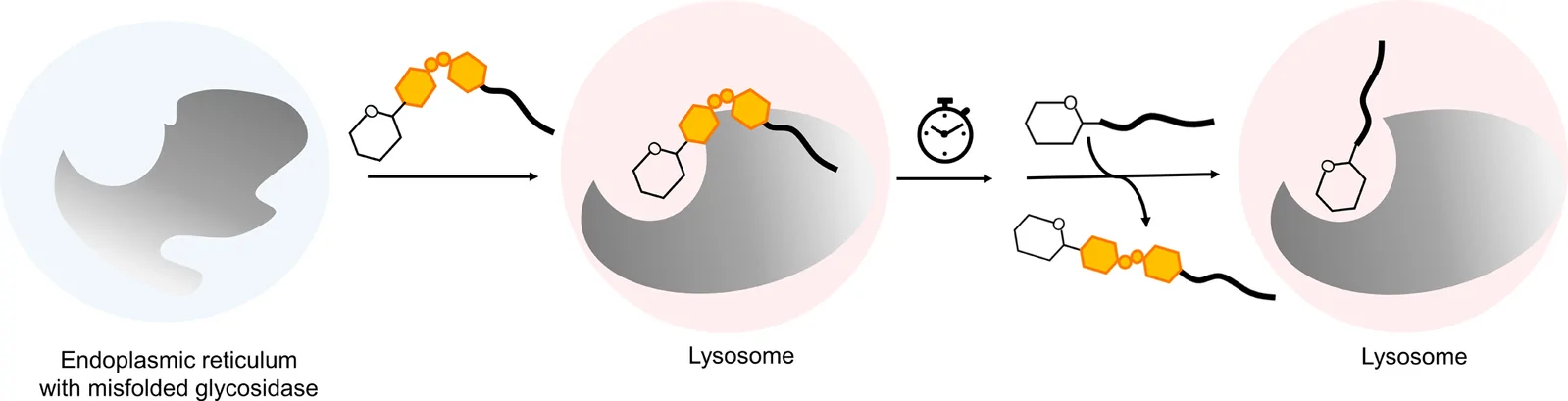
Use of ABGs as pharmacological chaperones. Here the *Z*-ABG (more potent than the *E* isomer) binds to a misfolded glycosidase in the endoplasmic reticulum (left) in order to restore the proper enzyme conformation. After trafficking to the lysosome, the stability of the complex decreases over time as the *Z*-ABG relaxes to its *E*-state, thus allowing binding of natural ligands.

Given these requirements, ABGs have emerged as promising candidates for next-generation PCs. However, the ubiquitous distribution of glycosidases throughout the human body complicates selective targeting, even when using light as an external stimulus. It is therefore preferable to design ABGs that exhibit maximal binding affinity in their metastable state, such that dissociation of the PC–enzyme complex can be coupled to the thermal relaxation process (Figure 18).

Recent studies have reported the synthesis and evaluation of ABGs as potential pharmacological chaperones (PCs) for glucosidase-related disorders, with particular emphasis on β-glucocerebrosidase (GCase). GCase catalyzes the hydrolysis of glucosphingolipids and is implicated in lysosomal storage disorders such as Gaucher disease (GD). Iminosugars and related scaffolds constitute the principal class of glucosidase inhibitors and have therefore served as privileged motifs for the design of corresponding ABGs.

For example, Cacciarini and co-workers developed mono- and divalent ABGs based on a trihydroxypiperidine core, varying both valency and linker length between the azobenzene unit and the glycomimetic ligand (Figure 19).^75,101^ The inhibitory potencies of compounds **58–59** toward GCase were evaluated in human leukocytes (Figure 19a). The results showed that activity increases with linker length, with the divalent analogue emerging as the most potent inhibitor. Notably, only these longer-linker systems fulfilled the requirements of light-controlled pharmacological chaperones, exhibiting higher activity in the *Z* configuration. Kinetic analyses supported by docking and molecular dynamics simulations suggested a mixed or noncompetitive mode of inhibition.

**Figure 19:**
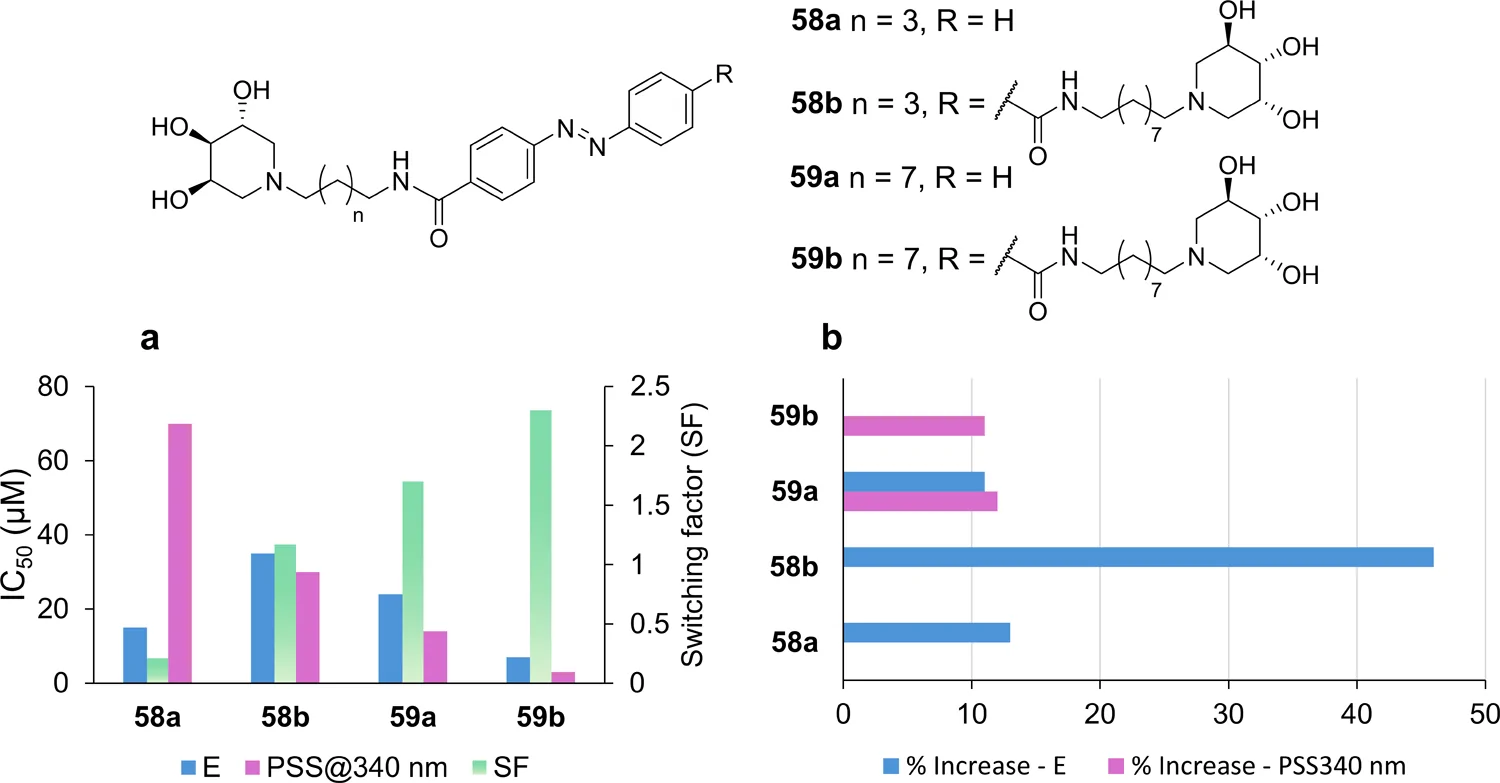
Photoswitchable pharmacological chaperones (PCs) based on trihydroxypiperidine ligands. (a) Comparison of IC_50_ values toward inhibition of human glucocerebrosidase (GCase) in the *E*-state and at PSS reached after irradiation with UV-light. The switching factor (SF) corresponds to the ratio IC_50_(*E*)/IC_50_(PSS). (b) Percentage enhancement of GCase activity in Gaucher disease fibroblasts. Here only compounds **59a** and **59b** were compared in *E*-state and at the UV-PSS.

Further biological evaluation of chaperone activity was carried out in fibroblasts derived from Gaucher disease patients, where restoration of GCase activity was measured in the presence of either *E*-configured or photoirradiated ABGs (Figure 19b). A modest enhancement (11%) was observed for the *Z* isomer of **59b**, whereas the *E* isomer of the shortest-linker analogue (**58b**) increased enzymatic activity by up to 46%. Despite encouraging results obtained with the single glycosidase, overall performance in more complex media such as fibroblasts remained limited, underscoring the difficulty of designing effective photoswitchable pharmacological chaperones and highlighting the need to better tune both binding affinity and the thermal relaxation kinetics of the *Z* isomer.

Further important contributions to the development of photoswitchable pharmacological chaperones for glycosidases have been reported by Xie, Ortiz Mellet, and García Fernández through the design of sp^2^-iminosugar-based ABGs.^53,72^ These systems exploit the pronounced sensitivity of sp^2^-iminosugar binding to both aglycone nature and orientation, making them particularly well suited for the incorporation of azobenzene photoswitches at structurally decisive positions.

A first library of sp^2^-iminosugar ABGs, based on 1-deoxynojirimycin (DNJ)^72^ and the bicyclic 5N,6O-oxomethylidenenojirimycin (ONJ),^53^ was prepared with systematic variation of linker architecture, spacer length, and valency. *para*,*para*′- and *ortho*,*ortho*′-DHAB scaffolds were functionalized either via mono-*O*-alkylation followed by glycosylation with ONJ pseudoglycosyl donors, or via double *O*-alkylation to introduce terminal isothiocyanate groups, enabling thiourea formation with DNJ and subsequent cyclization to iminothiazolidine ONJ surrogates.

Photophysical characterization in DMSO showed nearly quantitative *E* → *Z* photoisomerization for most derivatives, with thermal half-lives of the *Z* isomers ranging from approximately 2 to >24 h at 37 °C, thus compatible with enzymatic assays. The resulting libraries were evaluated against panels of α- and β-glucosidases, including human β-glucocerebrosidase (GCase), and analyzed in terms of switching factor (SF = *K_i_* (Z)/*K_i_* (E)) and selectivity index (SI = *K_i_* (α)/*K_i_* (β)). Among linker-free ONJ *O*-glycosides, a *p,p*′-DHAB octyl derivative exhibited strong nanomolar inhibition of GCase in the *Z* state, albeit with limited α/β selectivity and a modest switching factor.^53^ In contrast, within the thiourea-linked series, monovalent iminothiazolidine conjugates consistently displayed the most favorable balance of high SI and low SF, with *Z* isomers reaching nanomolar *K_i_* values.^72^ Octyl-substituted iminothiazolidine ABGs were therefore selected for detailed GCase studies, as they best combined strong *Z*-state preference with enhanced isoenzyme discrimination.^72^

Notably, an *o*,*o*′-DHAB analogue showed inverted isomer preference depending on the enzyme, with the *E* isomer being more potent toward human GCase, while the *Z* isomer was favored for bovine β-glucosidase, highlighting the critical role of aglycone topology in isoenzyme selectivity. Selected candidates were further evaluated at pH 7.3 and 5.5 to mimic endoplasmic reticulum and lysosomal environments. These compounds retained submicromolar *K_i_* values and moderate switching factors under both conditions, particularly at neutral pH, supporting their potential as photoswitchable pharmacological chaperones capable of light activation prior to trafficking and subsequent thermal deactivation in the lysosome (Figure 20).

**Figure 20:**
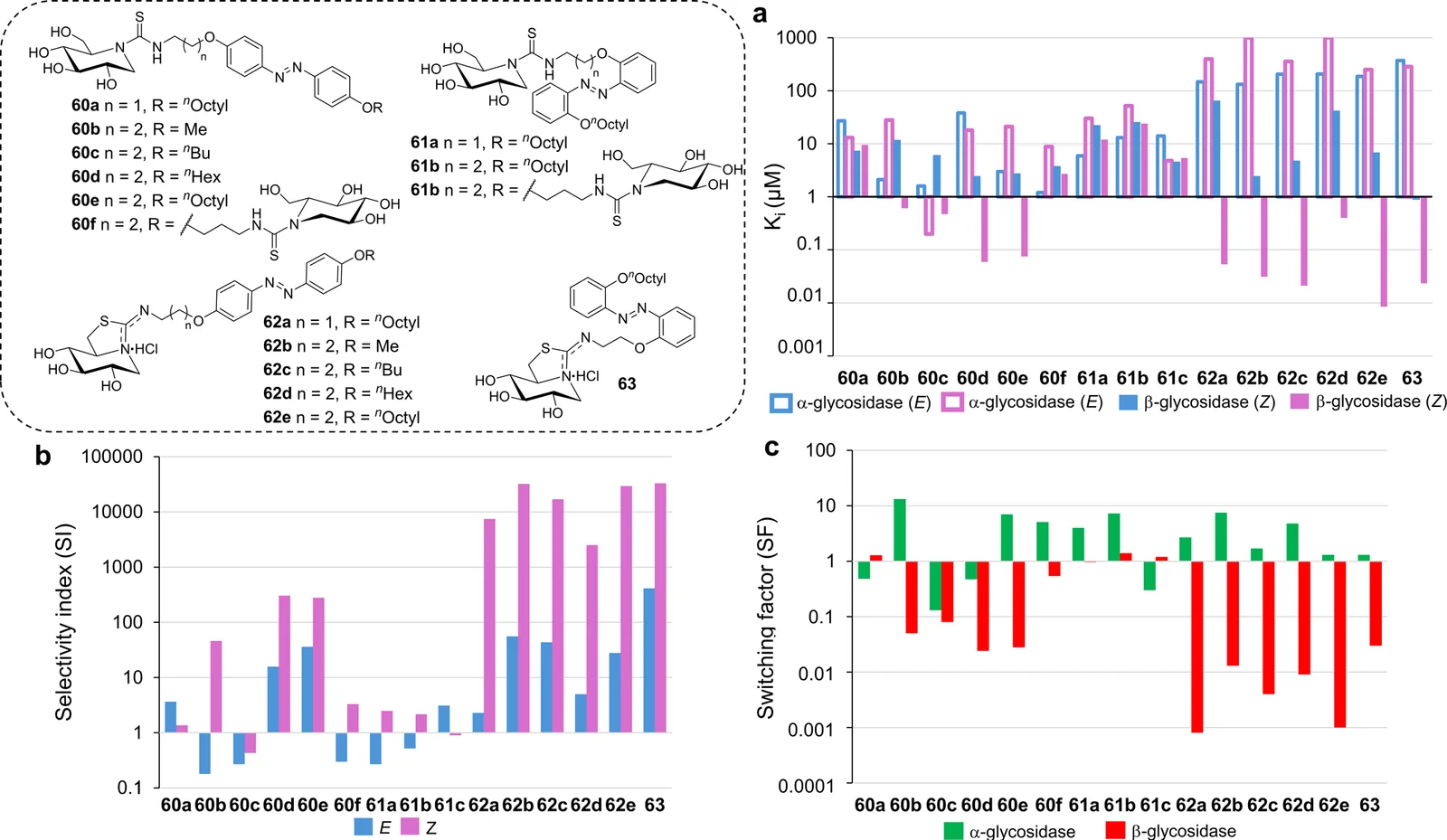
A Library of photoswitchable PCs based on sp^2^-imminosugar ligands. (a) Comparison of inhibitory constants (K_i_) measured in the *E*-state or *Z*-rich PSS for an α- and a β-glucosidase. (b) Comparison of selectivity indexes (SI) of ABGs in *E*- or *Z*-state; the SI is defined as the ratio K_i_(α)/K_i_(β) for a given isomeric state. (c) Comparison of switching factors (SF) for inhibition of α- or β-glucosidase. The vertical scale on the three bar diagrams is logarithmic.

Taken together, these studies establish sp^2^-iminosugar-based ABGs as a versatile platform for structure–activity-guided optoglycomics targeting glycosidases. By tuning the glycomimetic headgroup, azobenzene positioning, linker architecture, and valency, both *E*/*Z* switching behavior and α/β isoenzyme selectivity can be controlled, enabling nanomolar GCase inhibition with switching factors spanning several orders of magnitude. This high adjustability together with preferential inhibition in the Z state and thermally driven deactivation, positions these systems as promising photoswitchable pharmacological chaperones for Gaucher disease and as a general blueprint for light-addressable glycosidase modulators.

### 6.3 CD1d-NKT

ABGs derived from α-galactosylceramide exploit light-induced *E*/*Z* isomerization to modulate the CD1d−α-GalCer–TCR ternary complex and thereby regulate cytokine production by invariant NKT (iNKT) cells (Figure 21a). These systems are typically constructed by introducing an azobenzene unit into the acyl chain of KRN7000 analogues via amide bond formation, either at the terminal position or within the lipid chain. This yields α-GalACer derivatives that retain the α-galactose headgroup while incorporating a conformationally switchable hydrophobic tail.^71,74,102^

**Figure 21:**
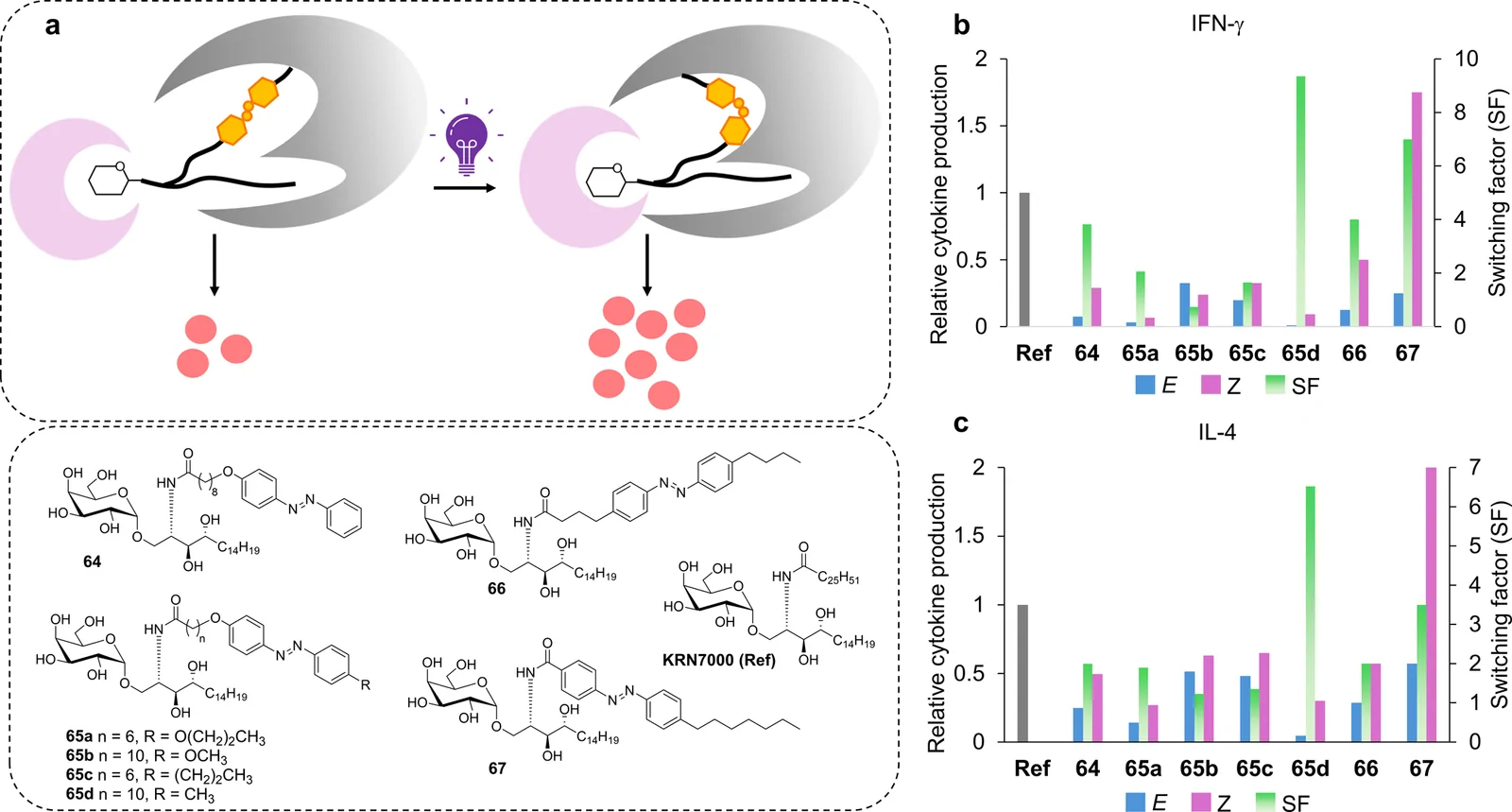
Photocontrol over of cytokines production with AB-based galactoceramide derivatives. (a) Modulation of cytokine production through photoswitching of CD1d-ABG-NKT ternary complex stability: the lipidic aglycon part of the ABG better fits into the pocket of CD1d in the *Z* state, thus leading to a closer proximity of the three partners and an increase in cytokine production. (b,c) Comparison of *E*- and *Z*-ABGs **64–67** toward IFN-g and IL-4 cytokines production, relative to the reference compound KRN7000. The switching factor (SF) is defined as the ratio between cytokine amount in *E* and *Z* states.

Overall, ABGs reported by the Yuasa and Trauner groups display efficient *E*/*Z* photoisomerization under UV/visible irradiation and thermally revert on experimentally convenient time scales. However, these photoglycolipids are prone to aggregation in aqueous media, which can impair photoisomerization efficiency, as noted by Yuasa and co-workers.^102^ Accordingly, their use requires concentrations below the critical aggregation concentration (CAC) or the addition of suitable surfactants.

In CD1d-binding assays, azobenzene placement within the lipid chain strongly influences photoregulation of antigen presentation. Terminal-chain substitution enables modest light-dependent control, with approximately 20% changes in affinity upon irradiation, highlighting the sensitivity of the CD1d lipid-binding groove to subtle changes in acyl-chain geometry.^71^ In contrast, positioning the azobenzene unit in the middle of the acyl chain significantly amplifies the switching response. In several α-GalCer analogues, even at low concentrations (≈10 ng mL^–1^), the *Z* isomers exhibit 3.2–6.8-fold higher activity than the corresponding *E* isomers in formation of the iNKT TCR−α-GalCer–CD1d ternary complex, accompanied by enhanced cytokine production in the *Z* state (Figure 21b,c).^71^

Consistent with this trend, cytokine ratios (Z/E) span 0.73–9.35 for IFN-γ and 1.23–6.52 for IL-4, although absolute cytokine levels remain 1.5–15-fold lower than those induced by KRN7000. In contrast, complementary azobenzene−α-GalCer designs can surpass the activity of the parent glycolipid in their photoactivated form; for example, compound **67** becomes more potent than KRN7000 upon 370 nm irradiation.^74^

Collectively, these galactosylceramide-based ABGs demonstrate that both the position and isomeric state of the azobenzene unit can be used to finely tune CD1d binding and downstream cytokine output. This enables reversible optical control over the CD1d–TCR axis and offers a promising strategy for modulating iNKT-cell responses and potentially mitigating anergy in NKT-cell-based immunotherapy.

### 6.4 Glycosylated Membrane Models

Light-induced photoisomerization provides a convenient external stimulus with which membrane rearrangements can be induced in a spatially and temporally controlled manner. To this end, azobenzene glycolipid mimetics have emerged as particularly attractive tools for probing membrane dynamics. Upon photoisomerization, the molecular geometry of the embedded glycolipid changes, thereby perturbing the surrounding membrane in a controlled and reversible manner. This way, light-induced molecular reorganization is translated into structural changes at the membrane level.

In particular, Lindhorst, Murphy, and co-workers developed a series of azobenzene glycoconjugates based on glycerolipid scaffolds bearing alkyl chains of 12–16 carbon atoms (Figure 22).^69,103^ These compounds were incorporated either into dipalmitoylphosphatidylcholine (DPPC) Langmuir monolayers or into lyotropic liquid-crystalline mixtures composed of DPPC and didecanoylphosphatidylcholine (DLPC).^104^

**Figure 22:**
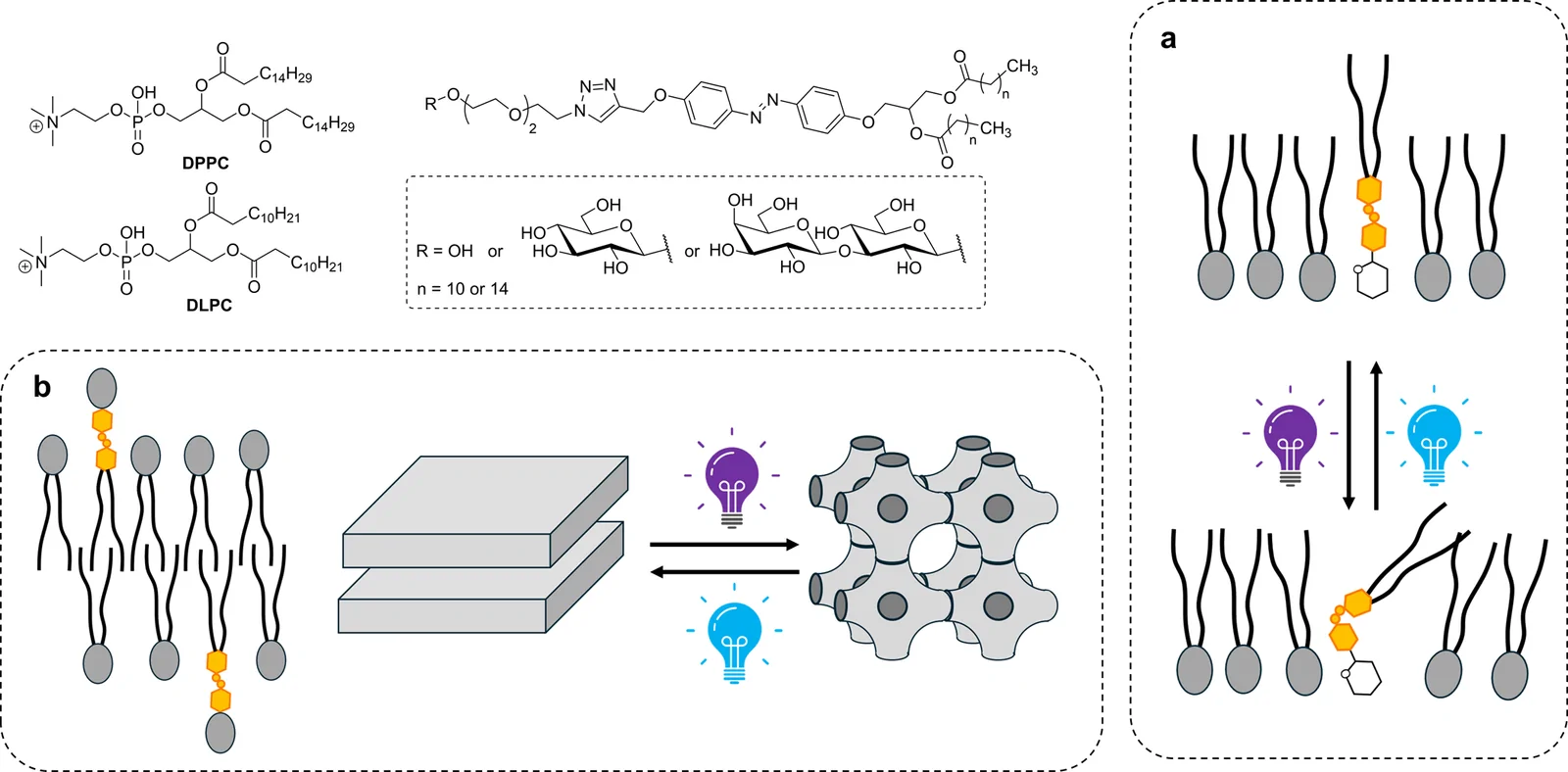
Photoswitchable glycolipids for dynamic membrane modifications. (a) Reversible change in orientation of lipidic ABGs embedded in monolayers of DPPC. (b) Lyotropic liquid crystals composed of DPPC, DLPC and photolipids show a reversible light-induced transition between a lamellar and a cubic mesophase. DPPC: dipalmitoylphosphatidylcholine; DLPC: didecanoylphosphatidylcholine.

Within DPPC monolayers, efficient photoswitching of the embedded ABGs was observed. Combining Langmuir compression isotherms with X-ray scattering measurements revealed that *E*/*Z* photoisomerization induces reorientation of the glycolipids within the monolayer, as reflected by changes in both surface pressure and film thickness (Figure 22a). When incorporated into lyotropic liquid crystals, photoisomerization likewise produced measurable structural rearrangements, as evidenced by SAXS and time-resolved SAXS experiments. Although the ABGs induced only subtle changes in the mesophase organization, a pronounced photoinduced phase transition was observed for the corresponding azobenzene lipid lacking a carbohydrate headgroup, which reversibly switched from a lamellar phase in the *E* state to a cubic phase in the *Z* state (Figure 22b).

Together, these studies demonstrate that azobenzene-containing glycolipids can couple molecular-scale photoisomerization to mesoscopic reorganization of membrane assemblies, providing valuable design principles for the development of light-responsive glycomaterials.

Trauner, Franquelim, and co-workers likewise investigated how photoswitchable lipids influence the reversible reorganization of model membranes.^70^ In this study, a series of azobenzene-containing sphingolipids, differing in both their polar headgroups, including glycosides, and sphingoid backbones, were incorporated into model membrane systems comprising small unilamellar vesicles (SUVs) and supported lipid bilayers (SLBs) (Figure 23).

**Figure 23:**
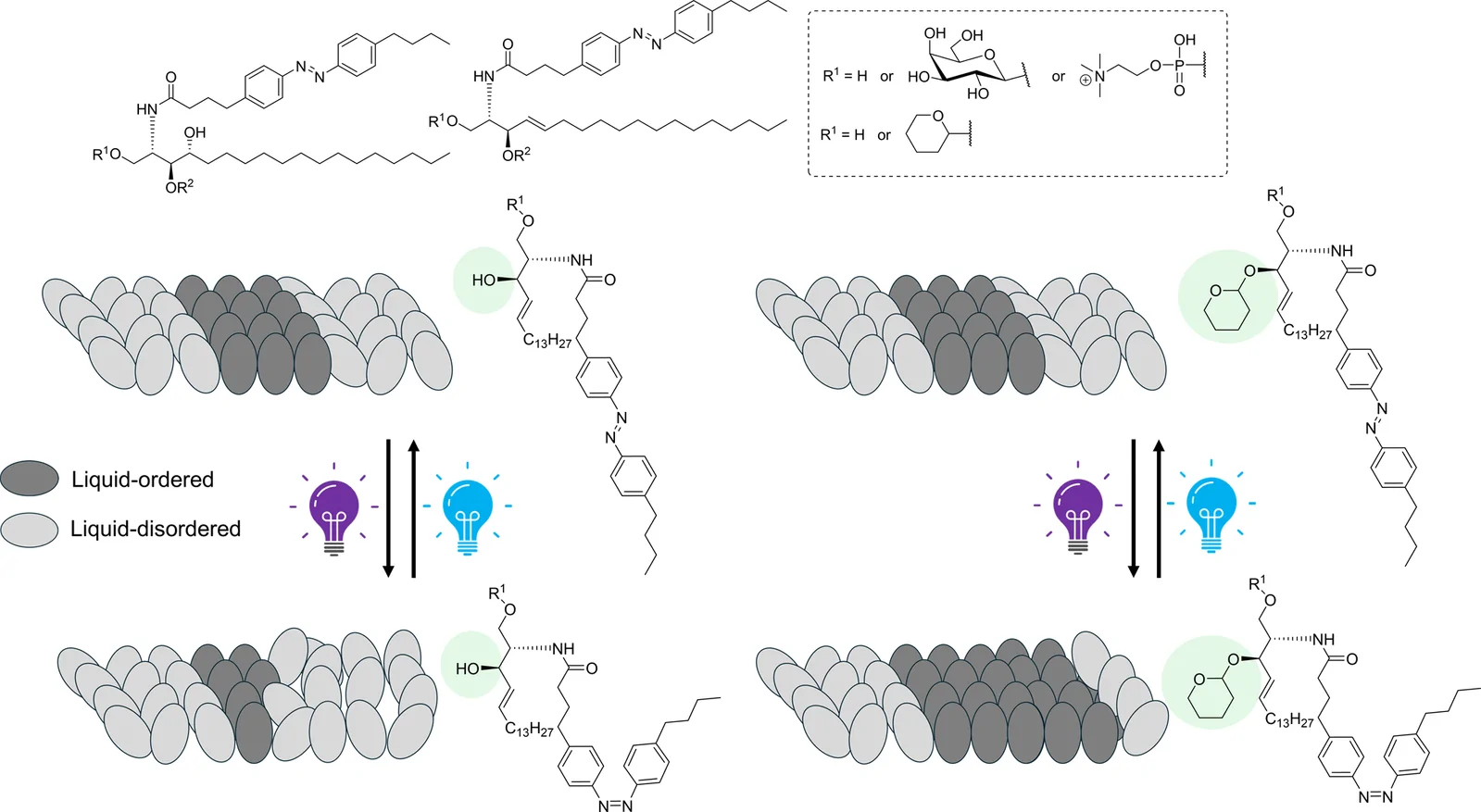
Photocontrolled membrane remodeling using AB-based sphingolipids. The phase transition outcome upon photoswitching mostly depends on the 3-OH group (free or THP-protected) on the sphingoid backbone.

Efficient and reversible *E*/*Z* photoisomerization was observed for the photolipids embedded in SUVs, indicating that the lipid environment does not impede photoswitching and supporting analogous behavior in the SLB model. Membrane remodeling upon irradiation was monitored by atomic force microscopy (AFM) and fluorescence microscopy, revealing reversible changes in phase-separated membrane domains. In particular, the *E* isomers preferentially partitioned into the more rigid liquid-ordered (L_o_) domains, whereas the *Z* isomers exhibited a greater affinity for the more fluid liquid-disordered (L_d_) regions of the membrane.

A key outcome of the study was that photoinduced membrane remodeling is governed primarily by the structure of the sphingoid backbone rather than by the nature of the headgroup. Specifically, photolipids bearing a free 3-hydroxyl group induced a decrease in the L_o_ phase upon *E* → *Z* photoisomerization, whereas the corresponding tetrahydropyranyl (THP)-protected analogues produced the opposite effect, increasing the area of the L_o_ domain in the *Z* state.

Collectively, these studies demonstrate that lipidic ABGs translate molecular photoisomerization into membrane-scale structural reorganization. Such optical control over membrane architecture is expected to provide new opportunities for regulating membrane-associated protein function, glycan presentation, and light-triggered drug delivery applications.

## 7 Future Perspectives of Optoglycomics

Glycobiology is characterized by extraordinary structural and functional complexity. Deciphering the biological roles of the glycome and translating this knowledge into diagnostic and therapeutic applications require methodologies that precisely manipulate glycan function in cells and, ultimately, living organisms. In recent years, light has emerged as a particularly attractive stimulus through the development of photoswitchable glycoconjugates, which combine carbohydrate ligands with chromophores that convert light into reversible structural changes. Owing to its exceptional spatial and temporal resolution, light provides unprecedented opportunities to study and control glycan-dependent biological processes under physiological conditions.

To describe this strategy of using photoswitchable glycoconjugates to investigate and manipulate carbohydrate recognition and glycan function, we introduced the term optoglycomics. Optoglycomic approaches should be distinguished from other light-based methodologies, such as photoaffinity labeling and photocaging. In optoglycomics, reversible light-induced structural changes of glycoconjugates are directly coupled to measurable biological responses, providing a conceptual framework for elucidating glycan function in model systems, at cell surfaces, and increasingly in living organisms.

To date, azobenzene has been the dominant molecular photoswitch in optoglycomics because of its favorable photochemical properties, synthetic accessibility, and compatibility with glycoconjugate design. A wide range of azobenzene-based architectures has enabled optical regulation of lectin binding, glycosidase inhibition, glycolipid recognition by invariant natural killer T cells through CD1d presentation, and glycan-mediated cell adhesion, in addition to structural reorganization of membrane models. Nevertheless, the field is expected to expand beyond azobenzene, paralleling developments in photopharmacology,^12,105^ with other classes of molecular photoswitches likely to play an increasingly important role in future optoglycomic applications.

A unique opportunity offered by optoglycomics is the ability to investigate how the spatial presentation and orientation of glycans within multivalent and supramolecular assemblies govern biological recognition. Photoswitchable glycointerfaces have shown that glycan presentation can be reversibly manipulated with light, enabling dynamically addressable biomaterials for selectively adhesive surfaces, patterned cell culture substrates, and, ultimately, immunologically active interfaces for regenerative medicine and xenotransplantation in the future.

Many molecular designs and biological applications remain to be explored. For example, the development of orthogonally addressable photoswitches raises the prospect of multiplexed glycointerfaces capable of independently controlling multiple glycan-mediated processes on a single surface. Another promising direction is the integration of optoglycomics with modern bioorthogonal chemistry. Copper-free strain-promoted azide–alkyne cycloaddition and related ligation strategies should facilitate the incorporation of photoswitchable glycoconjugates into living cells and organisms with minimal perturbation.

Looking further ahead, optical control of glycan metabolism itself represents an exciting frontier. Photoswitchable metabolic intermediates, glycosyltransferase substrates, or enzyme inhibitors could enable reversible regulation of glycoconjugate biosynthesis, including therapeutically relevant processes such as antibody glycosylation, with high temporal precision.

Beyond glycobiology, azobenzene glycosides are also beginning to find applications in other areas of biomedicine. Recently, azobenzene-bridged ionizable amphiphilic Janus glycosides were introduced as photoswitchable plasmid DNA carriers. Reversible *E*/*Z* photoisomerization modulated the supramolecular organization of the DNA nanocomplexes, thereby controlling transfection efficiency and, in vivo, organ-specific gene delivery.^106^

Despite the remarkable progress achieved to date, several challenges continue to limit the broader application of optoglycomics. At the molecular level, the incorporation of a photoswitch into a glycoconjugate adds an additional layer of complexity to glycoligand design. The conjugation of a photoswitch with the glycoligand can result in lectin binding differences, which are difficult to predict. Identifying the optimal conjugation site, as well as the appropriate linker length, flexibility, and geometry, remains largely empirical.

In addition, computer-assisted modeling of photoswitching of photosensitive glycoconjugates and its impact on carbohydrate–lectin recognition remains challenging. Consequently, rational design is frequently complemented by extensive synthetic optimization and experimental screening.

From a photochemical perspective, the properties of the photoswitch itself impose additional constraints. Incomplete photostationary states, the requirement for short-wavelength visible light, limited thermal stability of the metastable isomer, and insufficient fatigue resistance can all compromise biological applications. Furthermore, the development of orthogonal photoswitches that can be addressed independently with different wavelengths, a prerequisite for multiplexed optical control of several glycan-mediated processes, remains a formidable challenge.

In addition, optoglycoimoic applications are frequently complicated by the multivalence problem. Since the activity of a glycoligand often depends not only on the affinity of a single glycan but also on its multiple presentation and the spatial organization of multivalent glycoligands, a carbohydrate photoswitch may therefore also have to be designed as a multivalent glycoconjugate. Multivalent photoswitches on the other hand may influence each other such that the photochemical properties of the system are compromised through several photophysical effects. To predict the optimal distance, orientation, flexibility, or valence of a carbohydrate photoswitch makes structure–activity relationships in the glycosciences considerably more difficult to predict than for conventional small-molecule photopharmaceuticals.

Finally, although proof-of-principle studies increasingly demonstrate the potential of optoglycomics in living systems, applications in vivo are still in their infancy. As in photopharmacology more broadly, clinically relevant applications will require photoswitches that operate with tissue-penetrating wavelengths, exhibit favorable pharmacokinetic properties, and can be activated with high spatial precision in complex biological environments. Nevertheless, recently, rather simple azobenzene derivatives have been shown to restore vision in blinded zebrafish, with first-in-human clinical trials now underway.^107^

Despite its limitations, the future of optoglycomics appears highly promising. The field is poised to benefit from the rapid advances in photopharmacology, photomedicine, and optical technologies, including the development of red-shifted and near-infrared-responsive photoswitches, improved imaging methodologies, and miniaturized LED- and fiber-optic-based light-delivery systems capable of precise in vivo illumination. Together, these developments are expected to substantially expand the scope of optoglycomics, enabling increasingly sophisticated investigations of glycan function and, ultimately, opening new opportunities for the optical control of carbohydrate-mediated biological processes.

Although optoglycomics is still in its infancy, the studies discussed in this Perspective demonstrate that light provides a powerful, noninvasive means of interrogating and controlling glycan function with molecular precision. As photoswitch design, bioorthogonal chemistry, and glycoengineering continue to advance, optoglycomics is expected to emerge as an enabling technology for uncovering fundamental principles of glycobiology while opening new avenues for glycan-based diagnostics, biomaterials, and precision therapeutics.

## Notes

This work is dedicated to Prof. Joachim Thiem on the occasion of his 85th birthday.
